# TRIM25 enhances hypoxia signaling by catalyzing K11-linked polyubiquitination and stabilization of HIF-α

**DOI:** 10.1016/j.jbc.2026.113125

**Published:** 2026-05-06

**Authors:** Ziyi Li, Jun Li, Zhi Li, Rui Wang, Le Yuan, Yanan Song, Yanyi Wang, Runkun Yan, Fuxiang Lai, Jing Wang, Wuhan Xiao

**Affiliations:** 1 State Key Laboratory of Breeding Biotechnology and Sustainable Aquaculture, Institute of Hydrobiology, Chinese Academy of Sciences; Hubei Hongshan Laboratory, Wuhan, PR China; 2 Laboratory for Marine Biology and Biotechnology, Qingdao Marine Science and Technology Center, Qingdao, PR China; 3 University of Chinese Academy of Sciences, Beijing, PR China; 4 The Innovation of Seed Design, Chinese Academy of Sciences, Wuhan, PR China

**Keywords:** TRIM25, HIF-α, ubiquitination, hypoxia signaling

## Abstract

TRIM25 is an E3 ubiquitin ligase involved in various cellular processes due to its enzymatic activity. In particular, it plays a role in antiviral innate immunity. Here, we demonstrate that TRIM25 modulates hypoxia signaling. TRIM25 interacts with HIF-1α and HIF-2α, stabilizing them. TRIM25 catalyzes K11-linked polyubiquitination of HIF-1α at K719 and K721 and of HIF-2α at K709. This results in the stabilization of the proteins and enhanced hypoxia signaling. Moreover, TRIM25-mediated augmentation of hypoxia signaling depends on HIF-1α. *Trim25*-deficient mice are more sensitive to hypoxia, and zebrafish lacking *trim25* show a similar phenotype. These data reveal TRIM25’s role in regulating hypoxia signaling and provide insight into a new mechanism that modulates the stabilization and activity of HIF-1α and HIF-2α.

Oxygen (O_2_) is indispensable for the normal functioning and development of aerobic organisms (1). To adapt to varying oxygen levels, aerobic organisms have evolved multiple mechanisms that sense O_2_ levels and regulate physiological processes (1). HIF-1α and HIF-2α are two master regulators of hypoxia signaling whose transcriptional activity controls hypoxic physiological processes (2). The PHD/VHL system is a major regulatory mechanism for their function (2, 3). However, other modifications of HIF-1α and HIF-2α that regulate this pathway have also been identified, in particular ubiquitination and deubiquitination (4).

TRIM25 belongs to the tripartite motif (TRIM) protein family of E3 ubiquitin ligases, which contains a RING domain, two B-box domains, a coiled-coil domain and a C-terminal SPRY domain (5). TRIM25 is involved in various cellular processes, including cell proliferation, development, cancer progression, RNA binding, and antiviral activity (6).

One of the best-characterized roles of TRIM25 is to regulate RIG-I signaling by catalyzing K63-linked polyubiquitination of RIG-I (7). The K63-linked ubiquitin chains on RIG-I promote its interaction with mitochondrial antiviral signaling protein (MAVS) and subsequent downstream activation of intracellular antiviral signaling (7). TRIM25 also functions as an ISG15 E3 ligase to conjugate the ubiquitin-like protein ISG15 to target proteins (8), promoting TRIM25-mediated ISGylation (9). In addition to RIG-I, TRIM25 is also required for MDA5 and MAVS-mediated NF-κB activation and interferon production (10). The antiviral action of zinc-finger antiviral protein (ZAP), a cellular protein that inhibits viral mRNA translation, is enhanced by interaction with the SPRY domain of TRIM25 (11). TRIM25 can bind to RNA through its central CC domain (12). Therefore, it may influence intracellular signaling and/or replication of RNA viruses and use the RNA as a scaffold to get close to and modify its targets (13). TRIM25 can directly target the virus protein for proteasomal degradation to inhibit virus replication (14). On the other hand, some RNA viruses may use the capacity of TRIM25 to bind to RNA to inhibit its function (15). Furthermore, TRIM25 has been shown to associate with antiviral stress granules to enhance TRIM25’s ubiquitination activity towards multiple antiviral proteins (16). Recently, due to its RNA-binding ability, proton-activated TRIM25 has been found to mediate exogenous RNA surveillance (17).

In addition to its function in antiviral innate immune response, growing evidence has highlighted the multifaceted role of TRIM25 in controlling different aspects of tumorigenesis (18). TRIM25 is overexpressed in many human cancers and frequently correlates with poor patient survival (19). Moreover, TRIM25 also interferes with different forms of cell death, including apoptosis, pyroptosis, necroptosis, ferroptosis, and autophagy (6).

Herein, we found that TRIM25 enhances hypoxia signaling. Mechanistic studies suggest that TRIM25 binds to HIF-α to catalyze K11-linked polyubiquitination of HIF-α, resulting in stabilization of HIF-α, thereby enhancing hypoxia signaling.

## Results

### *TRIM25* enhances hypoxia signaling

**Figure 1:**
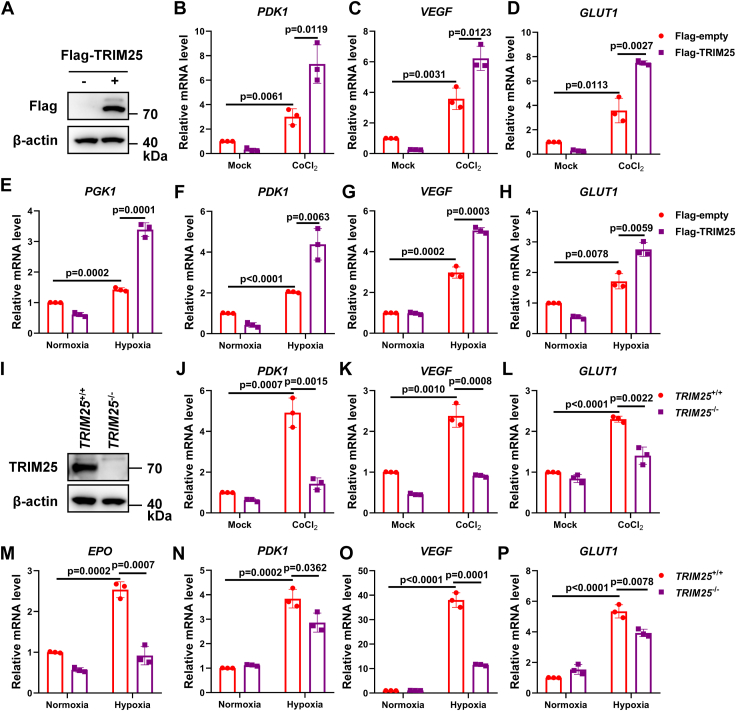
**TRIM25 enhances hypoxia signaling.***A*, immunoblotting (IB) of overexpressed Flag-*TRIM25* in U251 cells. *B*–*D*, quantitative real-time PCR (qPCR) analysis of *PDK1*, *VEGF*, and *GLUT1* mRNA in U251 cells transfected with Flag-empty vector or Flag-*TRIM25* for 12 h, followed by treatment with or without CoCl_2_ (200 μM) for 12 h. *E*–*H*, qPCR analysis of *PGK1*, *PDK1*, *VEGF*, and *GLUT1* mRNA in U251 cells transfected with Flag-empty vector or Flag-*TRIM25* for 8 h under normoxia (21% O_2_), followed by normoxia (21% O_2_) or hypoxia (1% O_2_) treatment for 16 h. *I*, IB of TRIM25 expression in wild-type (*TRIM25*^+/+^) and *TRIM25*-deficient (*TRIM25*^−/−^) U251 cells. *J*–*L*, qPCR analysis of *PDK1, VEGF*, and *GLUT1* mRNA in wild-type (*TRIM25*^+/+^) and *TRIM25*-deficient (*TRIM25*^−/−^) U251 cells treated with or without CoCl_2_ (200 μM) for 12 h. *M*–*P*, qPCR analysis of *EPO*, *PDK1, VEGF*, and *GLUT1* mRNA in wild-type (*TRIM25*^+/+^) and *TR*IM25-deficient (*TRIM25*^−/−^) U251 cells treated with normoxia (21% O_2_) or hypoxia (1% O_2_) for 16 h. Data in (*B*–*H*, *J*–*P*) are shown as mean ± SD; statistical significance was determined by unpaired two-tailed Student’s *t* test; each point represents a technical replicate of a representative experiment from three independent experiments.

*TRIM25* has been reported to be involved in several cellular processes (19, 20), but it is unknown whether it is involved in hypoxia signaling. We were interested in investigating whether *TRIM25* affects hypoxia signaling (4). When *TRIM25* was overexpressed in U251 cells, the typical hypoxia-responsive genes, including *PDK1*, *VEGF*, *GLUT1*, and *PGK1* (3), were dramatically up-regulated upon CoCl_2_ treatment or hypoxia treatment (Fig. 1, *A*–*H*). In contrast, the expression level of *PDK1*, *VEGF*, *GLUT1*, and *EPO* was significantly lower in *TRIM25*-deficient U251 cells compared to wild-type U251 cells when the cells were treated with CoCl_2_ (21) and hypoxia (Fig. 1, *I*–*P*) (22). Therefore, *TRIM25* may enhance hypoxia signaling.

### TRIM25 interacts with and stabilizes HIF-α

**Figure 2:**
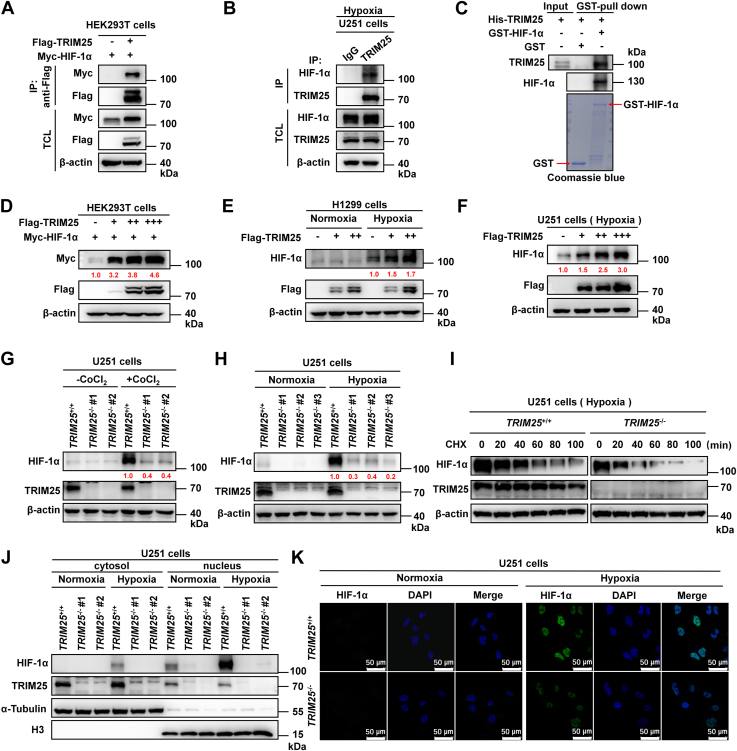
**TRIM25 interacts with HIF-1α to stabilize HIF-1α protein and cause HIF-1α accumulation in the nucleus.***A*, Co-IP of ectopically expressed TRIM25 interacting with ectopically expressed HIF-1α in HEK293T cells. *B*, endogenous IP of TRIM25 interacting with endogenous HIF-1α in U251 cells under hypoxia (1% O_2_) for 4 h. *C*, the interaction of bacterially expressed GST-HIF-1α and bacterially expressed His-TRIM25. *D*, IB of Myc-HIF-1α in HEK293T cells transfected with Myc-HIF-1α together with Flag-empty vector or increasing amounts of Flag-*TRIM25* expression plasmids. *E*, IB of endogenous HIF-1α in H1299 cells transfected with Flag-empty vector or increasing amounts of Flag-*TRIM25* expression plasmids for 18 h, followed by treatment with normoxia (21% O_2_) or hypoxia (1% O_2_) for 4 h. *F*, IB of endogenous HIF-1α in U251 cells transfected with Flag-empty vector or increasing amounts of Flag-*TRIM25* expression plasmids for 18 h, followed by treatment with hypoxia (1% O_2_) for 4 h. *G*, IB of endogenous HIF-1α in wild-type and *TRIM25*-deficient U251 cell lines (#1 and #2) treated with or without CoCl_2_ (200 μM) for 6 h. *H*, IB of endogenous HIF-1α in wild-type (*TRIM25*^+/+^) and *TRIM25*-deficient (*TRIM25*^−/−^) U251 cell lines (#1, #2 and #3) under normoxia (21% O_2_) or hypoxia (1% O_2_) for 4 h. *I*, IB of endogenous HIF-1α in wild-type (*TRIM25*^+/+^) and *TRIM25*-deficient (*TRIM25*^−/−^) U251 cell lines cultured under hypoxia (1% O_2_) for 4 h. CHX was then added to inhibit new protein synthesis with a final concentration of 50 μg/ml at the indicated time points. *J*, IB of endogenous HIF-1α in cytosolic and nuclear fractions in wild-type (*TRIM25*^+/+^) and *TRIM25*-deficient (*TRIM25*^−/−^) U251 cells cultured under normoxia (21% O_2_) or hypoxia (1% O_2_) for 4 h. *K*, Confocal microscopy image of endogenous HIF-1α in wild-type (*TRIM25*^+/+^) and *TRIM25*-deficient (*TRIM25*^−/−^) U251 cells under normoxia (21% O_2_) or hypoxia (1% O_2_) for 4 h. Scale bar = 50 μm.

To understand the underlying mechanisms by which *TRIM25* enhances hypoxia signaling, we examined whether TRIM25 interacts with HIF-1α. Overexpressed TRIM25 interacted with overexpressed HIF-1α as shown by co-immunoprecipitation assays (Fig. 2*A*). Under hypoxia, endogenous TRIM25 also interacted with endogenous HIF-1α (Fig. 2*B*). GST pull-down assays showed that bacterially expressed GST-HIF-1α interacted with bacterially expressed His-TRIM25 (Fig. 2*C*), suggesting that TRIM25 directly associates with HIF-1α.

The protein level of overexpressed HIF-1α was increased along with an increased amount of co-transfected TRIM25 (Fig. 2*D*). In both H1299 cells and U251 cells, the endogenous HIF-1α protein level was also increased under hypoxia, along with an increased amount of transfected TRIM25 (Fig. 2, *E* and *F*). In contrast, knockout of *TRIM25* caused a decrease of endogenous HIF-1α in U251 cells treated with CoCl_2_ and under hypoxia (Fig. 2, *G* and *H*) (22). When cycloheximide was added to block new protein synthesis (23), the endogenous HIF-1α decreased much more slowly in *TRIM25*^+/+^ U251 cells than in *TRIM25*^−/−^ U251 cells under hypoxia (Fig. 2*I*). Moreover, knockout of *TRIM25* mainly resulted in a decrease of HIF-1α in the nucleus under hypoxia (Fig. 2, *J* and *K*).

**Figure 3:**
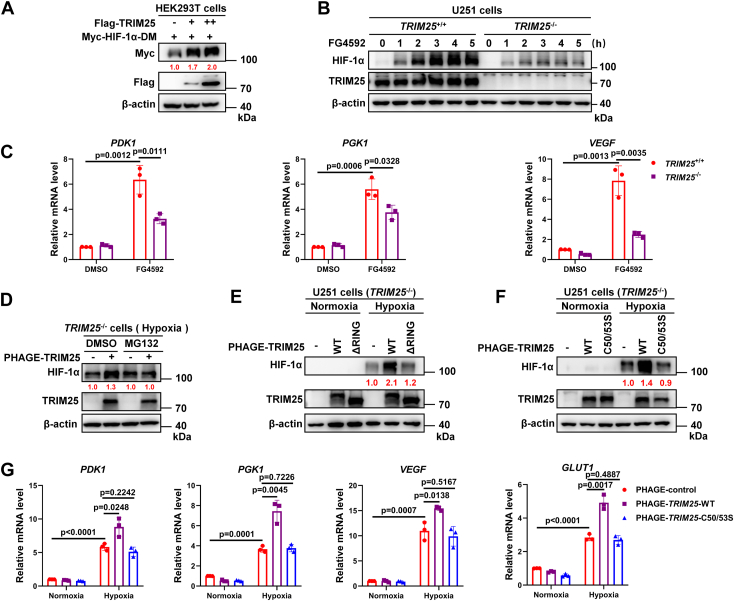
**TRIM25 causes HIF-1α stabilization dependent on its enzymatic activity, resulting in enhanced hypoxia signaling.***A*, IB of overexpressed Myc-HIF-1α-DM (encoding the double mutant of HIF-1α [P402A/P564A]) in HEK293T cells co-transfected with Flag-empty vector or increasing amounts of Flag-*TRIM25* expression plasmids. *B*, IB of endogenous HIF-1α in wild-type (*TRIM25*^+/+^) and *TRIM25*-deficient (*TRIM25*^−/−^) U251 cells treated with FG4592 (20 μM) for different time periods. *C*, qPCR analysis of *PDK1, PGK1* and, *VEGF* mRNA in wild-type (*TRIM25*^+/+^) and *TRIM25*-deficient (*TRIM25*^−/−^) U251 cells treated with DMSO or FG4592 (20 μM) for 12 h. *D*, IB of endogenous HIF-1α in *TRIM25*^−/−^ U251 cells reconstituted with PHAGE-control or PHAGE-*TRIM25* by lentivirus treated with DMSO or MG132 (20 μM) under hypoxia (1% O_2_) for 4 h. *E*, IB of endogenous HIF-1α in *TRIM25*^−/−^ U251 cells reconstituted with PHAGE-control, PHAGE-*TRIM25*-WT or PHAGE-*TRIM25*-ΔRING by lentivirus under normoxia (21% O_2_) or hypoxia (1% O_2_) for 4 h. *F*, IB of endogenous HIF-1α in *TRIM25*^−/−^ U251 cells reconstituted with PHAGE-control, PHAGE-*TRIM25*-WT or PHAGE-*TRIM25*-C50/53S by lentivirus under normoxia (21% O_2_) or hypoxia (1% O_2_) for 4 h. *G*, qPCR analysis of *PDK1, PGK1, VEGF*, and *GLUT1* mRNA in *TRIM25*^−/−^ U251 cells reconstituted with PHAGE control, PHAGE-*TRIM25*-WT or PHAGE-*TRIM25*-C50/53S by lentivirus under normoxia (21% O_2_) or hypoxia (1% O_2_) for 16 h. Data in (*C* and *G*) are shown as mean ± SD; statistical significance was determined by unpaired two-tailed Student’s *t* test; each point represents a technical replicate of a representative experiment from three independent experiments.

We then investigated whether the stabilization of HIF-1α by TRIM25 was dependent on the PHD/VHL pathway (2, 24). Overexpression of *TRIM25* still stabilized the HIF-1α proline mutant, HIF-1α-DM (in which the prolines 402 and 564 were mutated to alanine) (Fig. 3*A*). Furthermore, the addition of the specific proline hydroxylase inhibitor, FG4592 (23, 25), resulted in much higher HIF-1α protein levels in *TRIM25*^+/+^ U251 cells than in *TRIM25*^−/−^ U251 cells (Fig. 3*B*). Consistently, the expression of typical hypoxia-responsive genes *PDK1, PGK1*, and *VEGF* was higher in *TRIM25*^+/+^ U251 cells than in *TRIM25*^−/−^ U251 cells upon addition of FG4592 (Fig. 3*C*). These results imply that TRIM25-mediated stabilization of HIF-1α is independent of the PHD/VHL pathway.

Given that TRIM25 is a typical E3 ligase, we investigated whether the proteasome inhibitor MG132 could block the effect of TRIM25 on HIF-1α. Reconstitution of *TRIM25* in *TRIM25*^−/−^ U251 cells increased endogenous HIF-1α under hypoxia, but the addition of MG132 reduced this effect (Fig. 3*D*). We then determined whether the enzymatic activity of TRIM25 is required for TRIM25 to stabilize HIF-1α. The TRIM25 mutant lacking the RING domain (ΔRING), where the enzyme activation sites are localized, was unable to enhance HIF-1α like wild-type TRIM25 (Fig. 3*E*). Consistently, the enzyme-inactive mutant of TRIM25 (C50/53S) could not increase HIF-1α (Fig. 3*F*). As expected, reconstitution of wild-type TRIM25, but not of the enzyme-inactive mutant in *TRIM25*^−/−^ U251 cells, increased the expression of typical hypoxia-responsive genes, *PDK1, PGK1, VEGF*, and *GLUT1*, under hypoxia (Fig. 3*G*).

Taken together, these data suggest that TRIM25 directly interacts with HIF-1α to stabilize HIF-1α depending on the E3 ligase activity of TRIM25, leading to the enhancement of hypoxia signaling.

### TRIM25 targets the specific lysine residue (s) of HIF-α to catalyze the K11-linked polyubiquitination of HIF-α

**Figure 4:**
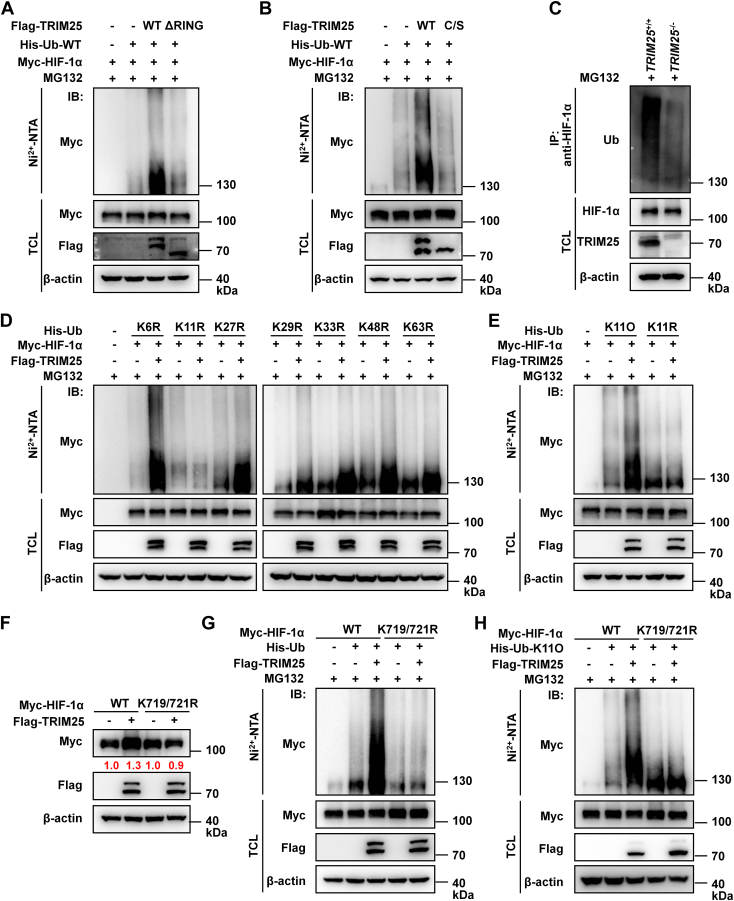
**TRIM25 catalyzes K11-linked polyubiquitination of HIF-1α at Lys719 and Lys721.***A*, IB of HIF-1α ubiquitination. HEK293T cells were transfected with Myc-HIF-1α, His-Ub-WT together with Flag-empty vector, Flag-TRIM25-WT or Flag-TRIM25-ΔRING expression plasmids for 20 h, then treated with MG132 (20 μM) for 4 h. *B*, IB of HIF-1α ubiquitination. HEK293T cells were transfected with Myc-HIF-1α, His-Ub-WT together with Flag-empty vector, Flag-TRIM25-WT or Flag-TRIM25-C/S (C50/53S) expression plasmids for 20 h, then treated with MG132 (20 μM) for 4 h. *C*, IB of endogenous HIF-1α ubiquitination in wild-type (TRIM25^+/+^) and TRIM25-deficient (TRIM25^−/−^) U251 cells treated with MG132 (20 μM) under hypoxia (1% O_2_) for 4 h. *D*, IB of HIF-1α ubiquitination. HEK293T cells were transfected with Myc-HIF-1α, Flag-empty vector or Flag-TRIM25 together with His-ubiquitin mutants (K6R, K11R, K27R, K29R, K33R, K48R, and K63R) expression plasmids for 20 h, then treated with MG132 (20 μM) for 4 h. *E*, IB of HIF-1α ubiquitination. HEK293T cells were transfected with Myc-HIF-1α, Flag-empty vector or Flag-TRIM25 together with His-Ub-K11O or K11R expression plasmids for 20 h, then, treated with MG132 (20 μM) for 4 h. *F*, IB of the indicated proteins in HEK293T cells transfected with Myc-HIF-1α-WT or its mutant Myc-HIF-1α-K719/721R together with Flag-empty vector or Flag-TRIM25 expression plasmids. *G*, IB of ubiquitination of HIF-1α and its mutant HIF-1α-K719/721R. HEK293T cells were transfected with His-Ub-WT, Myc-HIF-1α-WT or Myc-HIF-1α-K719/721R together with Flag-empty vector or Flag-TRIM25 expression plasmids for 20 h, then, treated with MG132 (20 μM) for 4 h. H, IB of ubiquitination of HIF-1α and its mutant HIF-1α-K719/721R. HEK293T cells were transfected with His-Ub-K11O, Myc-HIF-1α-WT or Myc-HIF-1α-K719/721R together with Flag-empty vector or Flag-TRIM25 expression plasmids for 20 h, then treated with MG132 (20 μM) for 4 h.

We then investigated whether TRIM25 catalyzes polyubiquitination of HIF-1α. When MG132 was added, overexpression of wild-type TRIM25 induced polyubiquitination of HIF-1α, whereas overexpression of the ΔRING and C50/53S mutants did not (Fig. 4, *A* and *B*). As expected, in the presence of MG132, polyubiquitination of endogenous HIF-1α was higher in *TRIM25*^+/+^ U251 cells than in *TRIM25*^−/−^ U251 cells (Fig. 4*C*). Further ubiquitination assays showed that TRIM25 catalyzed K11-linked polyubiquitination of HIF-1α (Fig. 4, *D* and *E*).

We then investigated which lysine residues of HIF-1α were targeted by TRIM25. First, we determined which region of HIF-1α was stabilized by overexpression of TRIM25 and found that the C-terminus of HIF-1α (400–826 aa) was targeted by TRIM25 for stabilization (Fig. S1*A*). Further assays showed that the mutants with either lysine 719 or lysine 721 mutated to alanine could not be stabilized by overexpression of TRIM25 (Figs. 4*F* and S1, *B* and *C*). The two lysine residues of HIF-1α are evolutionarily conserved between zebrafish and human (Fig. S1*D*). Overexpression of TRIM25 could induce K11-linked polyubiquitination of wild-type HIF-1α, but not of any of the single (K719R and K721R) and double mutants (K719/721R) (Figs. 4, *G* and *H* and S1*E*).

We knocked down HIF-1α using siRNA and found no difference in HIF-1α protein levels and the expression of its downstream target *PDK1* between *TRIM25*^+/+^ and *TRIM25*^−/−^ U251 cells (Fig. S2, *A* and *B*). This suggests that TRIM25's effect on hypoxia signaling depends on HIF-1α. Furthermore, overexpression of *TRIM25* in *TRIM25*^−/−^ U251 cells enhanced the expression of the typical hypoxia-responsive genes, including *PDK1*, *PGK1*, and *GLUT1*, activated by HIF-1α, whereas it had no significant effect on those activated by HIF-1a-K719/721R (Fig. S2*C*).

Next, we investigated whether TRIM25 has a similar effect on HIF-2α. Exogenous and endogenous TRIM25 bound to exogenous and endogenous HIF-2α (Fig. S3, *A* and *B*). Furthermore, under hypoxic conditions, the level of endogenous HIF-2α was lower in *TRIM25*^−/−^ U251 cells than in *TRIM25*^+/+^ U251 cells (Fig. S3*C*). Consistently, expression of the HIF-2α-regulated gene *PAI-1* was lower in *TRIM25*^−/−^ U251 cells than in *TRIM25*^+/+^ U251 cells under hypoxia (Fig. S3*D*).

An amino acid sequence comparison shows that K719 is the only residue conserved between HIF-2α and HIF-1α (Fig. S3*E*). Co-expression of TRIM25 stabilized the wild-type HIF-2α protein but not the mutant, HIF-2α-K709R (Fig. S3*F*). In the presence of MG132, TRIM25 induced K11-linked polyubiquitination of wild-type HIF-2α but not the mutant, HIF-2α-K709R (Fig. S3, *G* and *H*).

Taken together, these data suggest that TRIM25 targets K719 and K721 of HIF-1α, K709 of HIF-2α to catalyze K11-linked polyubiquitination, resulting in protein stabilization, thereby enhancing hypoxia signaling.

### TRIM25 facilitates hypoxia adaptation

To understand the biological consequence of TRIM25 in enhancing hypoxia signaling, we examined its role in cellular glucose uptake, lactate production, and ATP production, the main events related to hypoxia adaptation that are affected by hypoxia signaling (26, 27, 28, 29). Under hypoxia, reconstitution of *TRIM25* significantly promoted glucose uptake (Fig. S4, *A* and *B*). In contrast, the glucose uptake was lower in *TRIM25*^−/−^ U251 cells than in *TRIM25*^+/+^ U251 cells under hypoxia (Fig. S4, *C* and *D*). Similarly, reconstitution of *TRIM25* significantly enhanced lactate production under hypoxia (Fig. S4*E*). However, lactate production was lower in *TRIM25*^−/−^ U251 cells than in *TRIM25*^+/+^ U251 cells under hypoxia (Fig. S4*F*). Similar results were observed for ATP production (Fig. S4, *G* and *H*). Taken together, these data suggest that TRIM25 promotes glucose uptake, lactate production, and ATP production, thereby facilitating hypoxia adaptation.

### *Trim25*-deficient mice are more sensitive to hypoxia

To further investigate the function of *Trim25* in hypoxia signaling *in vivo*, we generated *Trim25*-deficient mice using CRISPR-Cas9 (Fig. S5, *A*–*C*). After intercrossing *Trim25*^+/−^ mice, *Trim25*^−/−^ mice were born in the expected Mendelian ratio. No gross defects were observed in *Trim25*^−/−^ mice, including body weight. As expected, the predicted mutant peptide (Trim25-Mut) produced in *Trim25*^−/−^ mice failed to interact with and stabilize endogenous HIF-1α compared to wild-type Trim25 (Fig. S5*D*). Consistently, overexpression of Trim25-Mut could not increase hypoxia-responsive gene expression under hypoxia compared to overexpression of wild-type *Trim25* (Fig. S5*E*).

**Figure 5:**
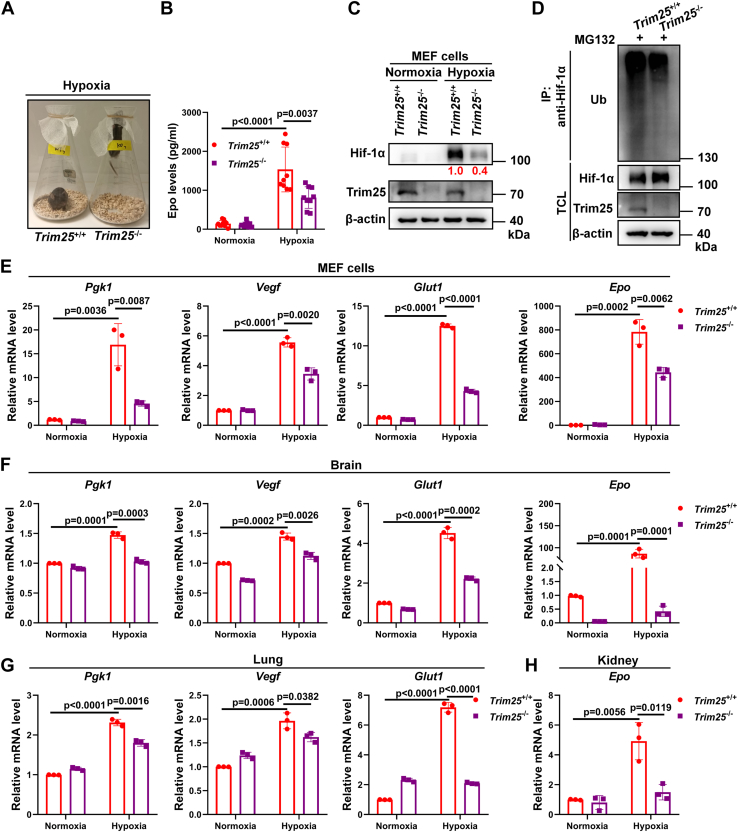
**Disruption of *Trim25* in mice attenuates hypoxia tolerance.***A*, images of *Trim25*^*+/+*^ and *Trim25*^*−/−*^ mice (8 weeks) exposed to hypoxia (10% O_2_) for 2 h. *B*, ELISA of Epo in serum from *Trim25*^*+/+*^ and *Trim25*^*−/−*^ mice (n = 9 per group) exposed to normoxia (21% O_2_) or hypoxia (10% O_2_) for 4 h. *C*, IB of endogenous Hif-1α in *Trim25*^*+/+*^ or *Trim25*^*−/−*^ MEF cells cultured under normoxia (21% O_2_) or hypoxia (1% O_2_) for 4 h. *D*, IB of endogenous Hif-1α ubiquitination in *Trim25*^*+/+*^ or *Trim25*^*−/−*^ MEF cells treated with MG132 (20 μM) and cultured under hypoxia (1% O_2_) for 4 h. *E*, qPCR analysis of *Pgk1, Vegf, Glut1*, and *Epo* mRNA in *Trim25*^*+/+*^ or *Trim25*^*−/−*^ MEF cells cultured under normoxia (21% O_2_) or hypoxia (1% O_2_) for 16 h. *F*, qPCR analysis of *Pgk1, Vegf, Glut1*, and *Epo* mRNA in the brain of *Trim25*^*+/+*^ or *Trim25*^*−/−*^ mice under normoxia (21% O_2_) or hypoxia (10% O_2_) for 4 h. *G*, qPCR analysis of *Pgk1, Vegf* and, *Glut1* mRNA in the lungs of *Trim25*^*+/+*^ or *Trim25*^*−/−*^ mice under normoxia (21% O_2_) or hypoxia (10% O_2_) for 4 h. *H*, qPCR analysis of *Epo* mRNA in the kidney of *Trim25*^*+/+*^ or *Trim25*^*−/−*^ mice under normoxia (21% O_2_) or hypoxia (10% O_2_) for 4 h. Data in (*B*, *E*–*H*) are shown as mean ± SD; statistical significance was determined by unpaired two-tailed Student’s *t* test; each point represents a technical replicate of a representative experiment from three independent experiments in (*E*–*H*).

To compare the hypoxia tolerance, we placed age-matched male *Trim25*^+/+^ and *Trim25*^−/−^ mice of similar weight in a hypoxia workstation (pre-set to 10% O_2_) and then observed their behaviors. After 2 h, *Trim25*^−/−^ mice started to jump to the gauze-sealed flask opening to breathe more oxygen, but *Trim25*^+/+^ mice appeared very calm, showing that *Trim25*^−/−^ mice were more sensitive to hypoxia (Fig. 5*A* and Video S1). Measurement of serum Epo showed that *Trim25*^−/−^ mice had lower Epo levels than *Trim25*^+/+^ mice (Fig. 5*B*). The protein level of Hif-1α in *Trim25*^−/−^ MEF cells was apparently lower than in *Trim25*^+/+^ MEF cells under hypoxia (Fig. 5*C*). Consistently, in the presence of MG132, polyubiquitination of endogenous Hif-1α was lower in *TRIM25*^−/−^ MEF cells than in *TRIM25*^+/+^ MEF cells (Fig. 5*D*).

We then compared the expression of typical hypoxia-responsive genes in MEF cells, brain, lung and kidney from *Trim25*^+/+^ and *Trim25*^−/−^ mice under hypoxia. The expression of *Pgk1, Vegf*, *Glut1*, and *Epo* was significantly lower in *Trim25*^−/−^ MEF cells than in *Trim25*^+/+^ MEF cells under hypoxia (Fig. 5*E*). Similar results were observed in brain, lung, and kidney (Fig. 5, *F*–*H*). These data suggest that disruption of *Trim25* in mice attenuates hypoxia tolerance.

### *Trim25*-deficient zebrafish are more sensitive to hypoxia

Since TRIM25 is evolutionarily conserved between human, mouse, and zebrafish, and the zebrafish model can provide clear phenotypes in hypoxia tolerance analysis (30, 31, 32), we used the zebrafish model to further evaluate the role of trim25 in hypoxia tolerance (Fig. S6*A*). First, we confirmed that zebrafish hif-1αa and hif-1αb interacted with zebrafish trim25, and trim25 stabilized hif-1αa and hif-1αb (Fig. S6, *B*–*E*). Furthermore, the hif-1αa mutant (K613/615R) and hif-1αb mutant (K672/674R), corresponding to the human HIF-1α mutant (K719/721R), could not be stabilized by trim25 (Fig. S6, *F* and *G*). Trim25 is highly expressed in the zebrafish brain and heart (Fig. S6*H*). We then used CRISPR-Cas9 to knock out *trim25* in zebrafish (Fig. S6, *I*–*L*).

**Figure 6:**
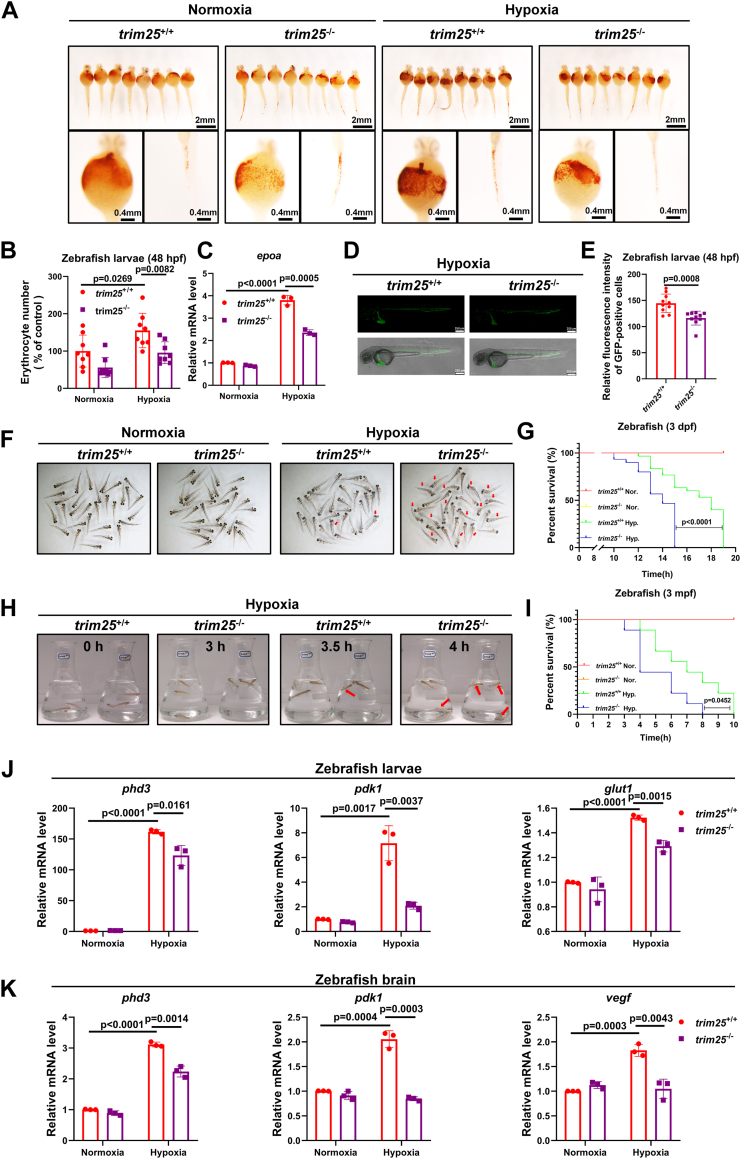
**Disruption of *trim25* in zebrafish attenuates hypoxia tolerance.***A*, O-dianisidine staining of functional hemoglobin in *trim25*^+/+^ and *trim25*^−/−^ zebrafish larvae after treatment with normoxia (21% O_2_) or hypoxia (10% O_2_) for 10 h. *B*, the number of erythrocytes was decreased in *trim25*^−/−^ zebrafish larvae compared to *trim25*^+/+^ zebrafish larvae after treatment with hypoxia (10% O_2_) for 10 h (n = 8 per group). *C*, qPCR analysis of *epoa* mRNA in *trim25*^+/+^ or *trim25*^−/−^ zebrafish larvae (3 dpf) under hypoxia (2% O_2_) for 6 h. *D*, fluorescence images of eGFP-labeled erythrocytes in *trim25*^+/+^ and *trim25*^−/−^ zebrafish larvae with Tg(gata1:eGFP) transgenic background after hypoxia (10% O_2_) exposure for 10 h. *E*, quantification of eGFP-labeled erythrocytes in (*D*) (n = 10 per group). *F*, images of *trim25*^*+/+*^ and *trim25*^*−/−*^ zebrafish larvae (3 dpf, n = 30 per group) exposed to normoxia (21% O_2_) or hypoxia (2% O_2_) for 12 h. The dead larvae (marked by red arrows) showed no movement, no blood circulation, and physical degeneration. *G*, survival curve of *trim25*^*+/+*^ and *trim25*^*−/−*^ zebrafish larvae (3 dpf, n = 30 per group) under normoxia (21% O_2_) or hypoxia (2% O_2_). *H*, images of *trim25*^*+/+*^ and *trim25*^*−/−*^ adult zebrafish (3 mpf, n = 3 per group) treated with hypoxia (2% O_2_) for 0, 3, 3.5, and 4 h. Red arrows indicate dying zebrafish. *I*, survival curve of *trim25*^*+/+*^ and *trim25*^*−/−*^ adult zebrafish (3 mpf, n = 9 per group) under normoxia (21% O_2_) or hypoxia (5% O_2_). *J*, qPCR analysis of *phd3, pdk1,* and *glut1* mRNA in *trim25*^+/+^ or *trim25*^−/−^ zebrafish larvae treated with normoxia (21% O_2_) or hypoxia (2% O_2_) for 6 h. K qPCR analysis of *phd3, pdk1,* and *vegf* mRNA in the brain of *trim25*^+/+^ or *trim25*^−/−^ adult zebrafish (3 mpf) treated with normoxia (21% O_2_) or hypoxia (5% O_2_) for 3 h. Data in (*G* and *I*) were determined by the Kaplan-Meier method and log-rank test. Data in (*B*, *C*, *E*, *J*, and *K*) are shown as mean ± SD; statistical significance was determined by unpaired two-tailed Student’s *t* test; each point represents a technical replicate of a representative experiment from three independent experiments in (*C*, *J*, and *K*).

O-dianisidine staining showed that the number of erythrocytes was lower in *trim25*^−/−^ zebrafish larvae than in *trim25*^+/+^ zebrafish larvae under hypoxia (Fig. 6, *A* and *B*). Consistently, the expression of *epoa* was lower in *trim25*^−/−^ zebrafish larvae than in *trim25*^+/+^ zebrafish larvae under hypoxia (Fig. 6*C*). *Gata1*-GFP-labeled zebrafish larvae also confirmed that the number of erythrocytes was lower in *trim25*^−/−^ zebrafish larvae than in *trim25*^+/+^ zebrafish larvae under hypoxia (Fig. 6, *D* and *E*).

We then compared the survival of *trim25*^−/−^ zebrafish and *trim25*^+/+^ zebrafish under hypoxia. For both larval and adult zebrafish, hypoxia treatment caused more deaths in *trim25*^−/−^ zebrafish than in *trim25*^+/+^ zebrafish (Fig. 6, *F*–*I* and Video S2). The expression of hypoxia-responsive genes, *phd3, pdk1, glut1* or *vegf* in zebrafish larvae and adult brain under hypoxia was lower in *trim25*^−/−^ zebrafish than in *trim25*^+/+^ zebrafish (Fig. 6, *J* and *K*).

Taken together, these data suggest that disruption of trim25 in zebrafish attenuates hypoxia tolerance.

## Discussion

As an E3 ligase, TRIM25 exerts its function mainly by catalyzing its targets for either ubiquitination or ISGylation. As the master regulators of hypoxia signaling, HIF-1α and HIF-2α are regulated by multiple post-translational modifications (33). Ubiquitination and deubiquitination have been widely identified and well defined to modulate the function of HIF-1α and HIF-2α, either enhancing or attenuating, in particular, their PHD/VHL-mediated oxygen-dependent proteasomal degradation (2, 24). Interestingly, in the TRIM family, TRIM44 is the only member that acts as a deubiquitinase identified to stabilize HIF-1α (34). However, it is still unknown whether the widely studied E3 ligase in the TRIM family, TRIM25, influences hypoxia signaling. In this study, from the increase in hypoxia-responsive gene expression under hypoxia after overexpression of TRIM25, we gradually found that TRIM25 binds to and catalyzes K11-linked polyubiquitination at K719 and K721 of HIF-1α and K709 of HIF-2α, leading to the stabilization of HIF-1α and HIF-2α protein levels, thereby enhancing hypoxia signaling and facilitating hypoxia tolerance. Therefore, this study reveals a previously unknown function of TRIM25 in hypoxia signaling.

Due to the seven lysine residues contained in ubiquitin, seven types of lysine-linked homogeneous polyubiquitination have been identified to modify target proteins, of which five types are considered to be non-canonical protein ubiquitination, with the exception of K48- and K63-linked polyubiquitination (35). K48-linked ubiquitination is known to mediate proteasomal degradation of target proteins, but other types of ubiquitination cause either degradation or stabilization of their target proteins (35). Although K11-linked polyubiquitination plays a role in the DNA damage response, this atypical ubiquitination is generally associated with the regulation of protein degradation. K11-linked ubiquitination of NOXA, β-TrCP1, SOX, and Ci promotes their degradation, whereas that of β-catenin leads to protein stabilization (36, 37, 38, 39, 40). For HIF-1α and HIF-2α, apart from K48- and K63-linked ubiquitination, other atypical forms of ubiquitination have hardly been identified (41, 42). Here, we found that TRIM25 catalyzes K11-linked polyubiquitination of HIF-1α and HIF-2α, resulting in their protein stabilization. This study uncovers a previously unrecognized atypical ubiquitination of HIF-1α and HIF-2α that leads to stabilization rather than degradation of HIF-1α and HIF-2α.

It is well-defined that HIF-1α and HIF-2α are stabilized under hypoxia due to the loss of enzymatic activity of the proline hydroxylases (2, 43). Here, we show that in the presence of TRIM25, HIF-1α and HIF-2α are more stable, suggesting that other factors can further regulate HIF-α′s stability even under hypoxia. However, to what extent this modulation contributes to hypoxia signaling is still hard to determine. Perhaps, compared to normoxia, this modulation under hypoxia is relatively mild, but still affects hypoxia signaling, leading to the influence on hypoxia adaptation and tolerance.

In particular, it is still uncertain whether the enhancement or attenuation of hypoxia signaling is beneficial for hypoxia tolerance. In fact, there are two categories of HIF target genes: one category of genes is related to hypoxia adaptation, such as *VEGF, EPO, LDHA*, *etc.*, and another category of genes is related to hypoxia-induced apoptosis, such as *BNIP3, NIX*, *etc.*, which may have opposite effects on hypoxia tolerance. Maintaining an appropriate balance between these two categories of genes may be critical for hypoxia tolerance. Therefore, enhancing or attenuating hypoxia signaling in a certain range after disruption of some genes may promote hypoxia tolerance *in vivo*. As reported previously, the different natural mutations identified in either *HIF-2α* or *PHD2* (*EGLN1*) in both animals and humans adapted to high altitude (hypoxia) show either increased or decreased hypoxia signaling (44). Here we have shown that disruption of TRIM25 attenuates hypoxia signaling and reduces hypoxia tolerance.

In summary, in this study, we found that TRIM25 targets HIF-1α and HIF-2α to stabilize their protein levels, resulting in the enhancement of hypoxia signaling. However, since multiple post-translational modifications of HIF-1α and HIF-2α can affect their activity, both TRIM25 and HIF-α are involved in cancer progression. To further investigate whether TRIM25-mediated HIF-α ubiquitination can affect other modifications of HIF-α and influence tumorigenesis will open a new window to fully understand the physiological functions of TRIM25 as well as the hypoxia signaling pathway.

### Experimental procedures

#### Ethics statement

The laboratory animal facility was accredited by the Association for Assessment and Accreditation of Laboratory Animal Care International (AAALAC), and all the animal protocols used in this study were approved by the Institutional Animal Care and Use Committee (IACUC) of the Institute of Hydrobiology, Chinese Academy of Sciences.

## Reagents and antibodies

See Table S1.

## Mice

*Trim25*^*−/−*^ mice on the C57BL/6 background were obtained from Cyagen Biosciences (https://www.cyagen.com/cn/zh-cn/sperm-bank-cn/S-KO-05246). Mice were maintained in isolated, ventilated cages (maximum of 5 mice per cage) in a barrier facility on a 12/12-h light/dark cycle, 22 to 26 °C, with unrestricted access to standard chow and tap water. *Trim25*^*−/−*^ mice were crossed with C57BL/6 mice to generate heterozygous mice (*Trim25*^+/−^). The wild-type (*Trim25*^+/+^) and homozygous (*Trim25*^−/−^) mice produced by mating heterozygous (*Trim25*^+/−^) mice with heterozygous (*Trim25*^+/−^) mice were used for further experiments. Eight-week-old mice were used in the experiments. Same-sex littermates were randomly assigned to the experimental groups.

### Zebrafish

The zebrafish strain AB and the transgenic line Tg(*gata1*:eGFP) (provided by Tingxi Liu, Shanghai Institutes for Biological Sciences, Chinese Academy of Sciences, China) were bred, maintained and staged according to standard protocols. *trim*25 knockout zebrafish were generated using CRISPR-Cas9 technology. The target site sequence 5′-GGATGCCTATAGAGTAAAAG-3′ was designed using http://zifit.partners.org/ZiFiT/. To obtain single guide RNA (sgRNA) targeting zebrafish *trim25*, pUC9 gRNA vector was used to amplify sgRNA template, and Transcript Aid T7 high yield transcription kit (Thermo Scientific) was used to synthesize and purify sgRNA. sgRNAs were co-injected with cas9 protein into wild-type zebrafish (strain AB) embryos at the one-cell stage. Eight injected embryos and uninjected embryos selected randomly were used as templates for genotyping by heteroduplex mobility assay (HMA). If the injected embryos had additional bands compared to the major bands of the uninjected embryos on the native PAGE, the remaining unidentified zebrafish embryos were reared as F0 generation. Adult zebrafish from the F0 generation were backcrossed to wild-type zebrafish to produce the F1 generation. If HMA patterns of the partial F1 generation were positive and effective editing of the target gene was confirmed by reading sequencing peak plots, the remaining F1 embryos were reared to adults for genotyping. F1 heterozygotes were crossed with the identical genotype (carrying the target mutation) to produce F2. The F2 generation contains wild-type (+/+), heterozygous (+/−), and homozygous (−/−) individuals in a ratio of 1:2:1. The primers used for genotyping were as follows: forward primer 5′-TCTGCGCCAATTGTCTCGAA-3′; reverse primer 5′-GGCCTCACATGATCTTCACA-3′. Finally, we obtained a mutant named *trim25* ^ihblzy01/ihblzy01^ (https://zfin.org/ZDB-ALT-240708-7).

### Cell culture

HEK293T and H1299 cells originally obtained from the American Type Culture Collection (ATCC), and U251 cells originally obtained from the Chinese Academy of Sciences were cultured in Dulbecco’s modified Eagle’s medium (DMEM) (VivaCell) supplemented with 10% fetal bovine serum (FBS). Primary mouse embryonic fibroblasts (MEFs) were prepared from embryos at 12.5 to 14.5 embryonic days and cultured in DMEM containing 10% FBS and 1% penicillin-streptomycin. All of the above cells were incubated at 37 °C in a 5% CO_2_ humidified incubator.

#### Quantitative real-time PCR (qPCR)

Total RNA was extracted using TransZol Up (TransGen Biotech) according to the manufacturer’s protocol. cDNA was synthesized using TransScript One-Step gDNA Removal and cDNA Synthesis SuperMix (TransGen Biotech). Quantitative real-time PCR (qRT-PCR) assays were performed using MonAmp SYBR Green qPCR Mix (High Rox) (Monad Bio.). Primers used for qRT-PCR are listed in Table S2.

#### Co-immunoprecipitation assay

Harvested cells were washed with PBS and lysed in 1 ml RIPA lysis buffer (50 mM Tris pH 7.4, 1% Nonidet P-40, 0.25% sodium deoxycholate, 1 mM EDTA pH 8.0, 150 mM NaCl, 1 mM NaF, 1 mM PMSF, 1 mM Na3VO4, and a 1:100 dilution of protease inhibitor mixture) for 30 min at 4 °C. Cell debris was removed by centrifugation (12,000*g*, 15 min) and 50 μl supernatant was used for total target protein detection. Anti-Flag antibody-conjugated agarose beads (Yeasen) were added to the remaining supernatant for overnight incubation at 4 °C. The precipitates were washed three times with lysis buffer, then mixed with β-mercaptoethanol and SDS loading buffer and boiled for 8 min. Protein analysis was performed by Western blot. The blots were photographed using a Fuji Film LAS4000 mini-luminescence image analyzer. ImageJ software was used to quantify protein levels based on the band density obtained by Western blot. For endogenous co-immunoprecipitation, Protein G Sepharose 4 Fast Flow (#17061801, Cytiva) and anti-TRIM25 primary antibody (A12938, Abclone) were used. Anti-rabbit IgG was used as a control.

## GST pull-down

The plasmids pGEX-4T-1, pGEX-4T-1-HIF-1α, and pET-32a-TRIM25 were transformed into *Escherichia coli* BL21 competent cells, respectively, and then induced with 0.5 mM IPTG at 16 °C with shaking at 200 rpm overnight. Bacteria were resuspended in PBS after centrifugation, and lysozyme (1 mg/ml) and protease inhibitor were added for 30 min on ice. The suspension was then lysed by sonication. GST and GST-HIF-1α recombinant proteins were separately bound to GST resin overnight at 4 °C, and then the precipitates were washed three times with PBS. His-TRIM25 recombinant protein was then added. The interaction of GST-HIF-1α with His-TRIM25 *in vitro* was detected by immunoblotting with anti-TRIM25 antibody. GST and GST-HIF-1α proteins were stained with Coomassie Blue.

### CRISPR-Cas9 knockout cell lines

Double-stranded oligonucleotides targeting *TRIM25* were annealed and cloned into Lenti-CRISPRv2 plasmids. The target sequence is listed in Table S1. Lenti-CRISPRv2-*TRIM25* sgRNA plasmids were then co-transfected with viral packaging plasmids (psPAX2 and pMD2G) into HEK293T cells. Six hours after transfection, the medium was changed. Viruses were harvested 2 days after transfection. The harvested viruses were ultrafiltered (0.45 μm filter; Millipore) and used to infect U251 cells. Puromycin was added to the culture medium at a final concentration of 1 μg/ml to screen for positive cells. After puromycin selection, we digested the cells into single cells, diluted them, and seeded approximately 50 cells per 10 cm dish. After allowing them to grow for 2 weeks to form clones, we randomly picked multiple single-cell clones using pipette tips and separately seeded them into 24-well plates. After a few days, we performed a Western blot to assess *TRIM25* knockout efficiency at the protein level in each well. Finally, three monoclonal cell lines with effective *TRIM25* knockout were retained and designated *TRIM25*^−/−^ #1, *TRIM25*^−/−^ #2, and *TRIM25*^−/−^ #3. These were derived from distinct single-cell clones picked with different pipette tips, representing three independent monoclonal cell lines generated by transducing a single sgRNA.

#### RNA interference

HIF-1α knockdown was performed by RNA interference. siRNA oligonucleotides targeting HIF-1α were synthesized in GenePharma Company. The sense sequence of HIF-1α-siRNA is: 5′-CCGUAUGGAAGACAUUAAATT-3′. HIF-1α-siRNA and negative control (NC) were transfected into U251 cells using Lipofectamine 2000 (Invitrogen) according to the manufacturer’s protocol. At 48 h post-transfection, HIF-1α protein level and hypoxia-responsive gene mRNA levels were detected.

### Nucleus and cytoplasm separation

Cells were cultured under normoxia (21% O_2_) or hypoxia (10% O_2_) for 4 h. The nuclear and cytoplasmic components were then separated using the Nuclear and Cytoplasmic Extraction Kit (#78833, Thermo Scientific) according to the manufacturer’s protocol. α-Tubulin was used as a cytoplasmic protein loading control, and histone H3 was used as a nuclear protein loading control.

### Immunofluorescence confocal microscopy

*TRIM25*^+/+^ and *TRIM25*^−/−^ U251 cells grown on glass Petri dishes were washed once with PBS and then fixed with 4% paraformaldehyde for 20 min at room temperature. Cells were washed five times with PBS and blocked with blocking buffer for 10 min. Cells were then incubated with anti-HIF-1α primary antibody (1:1000 dilution, Cell Signaling Technology) overnight at 4 °C, followed by five washes with PBS (containing 1% BSA). The cells were then incubated with secondary antibody (#A11008, Invitrogen) for 1 h at room temperature. Then, the cells were washed five times with PBS (containing 1% BSA). Finally, DAPI was used to counterstain the nucleus for 15 min, and the samples were washed five times with PBS (containing 1% BSA). Fluorescence microscopy images were captured with a fluorescence confocal microscope (SP8; Leica).

### Ubiquitination assay

HEK293T cells were transfected with indicated plasmids for 24 h. Denaturing buffer (6 M guanidine-HCl, 0.1 M Na_2_HPO4/NaH_2_PO4, 10 mM imidazole) was used to lyse the cells. Ubiquitination assays with His-ubiquitin or His-ubiquitin mutants were performed by affinity purification on Ni^2+^-NTA resin (Novagen). Anti-Myc antibody was used to detect polyubiquitination of Myc-HIF-1α. For the endogenous ubiquitination assay, cells were lysed with 100 μl 1% SDS at 100 °C for 5 min and sonicated on ice for 2 min. Then 900 μl RIPA with protease inhibitor was added, and the mixture was centrifuged at 4 °C for 15 min. Protein G Sepharose 4 Fast Flow (#17061801, Cytiva) and anti-HIF-1α primary antibody (D1S7W; Cell Signaling Technology) were used. Anti-ubiquitin antibody was used to detect ubiquitination of endogenous HIF-1α. In this assay, we used a series of ubiquitin mutants, including K-to-R (loss-of-function) and K-only (gain-of-function) mutants. For example, K11R means that only K11-linked polyubiquitin chains cannot be formed, whereas the other six types of polyubiquitin chains are formed normally. K11O refers to a ubiquitin molecule in which only the K11 residue is retained, while the lysine residues at the other six lysine sites are mutated to arginine. Therefore, the K11O mutant can only form K11-linked polyubiquitin chains and cannot form the other six types of polyubiquitin chains.

### Glucose uptake

Cells were pretreated with DMEM without glucose (PM150270, Pricella). The cells were then cultured under normoxia (21% O_2_) or hypoxia (1% O_2_) for 3 h. 2-NBDG (#18669-07-6, MCE) was added to the cell culture medium at a final concentration of 50 μM. Cells were further cultured under normoxia (21% O_2_) or hypoxia (1% O_2_) for 0.5 h in the dark. The ability of cells to take up fluorescent glucose analog 2-NBDG was measured by flow cytometry analysis.

### Lactate production measurement and ATP production measurement

Cells were incubated under normoxia (21% O_2_) or hypoxia (1% O_2_) conditions for 24 h. Then, cell culture medium was harvested for the measurement of lactate production using the L-Lactic Acid (L-LA) Content Assay Kit (AKAC001C, Boxbio). Cells were harvested for the measurement of ATP production using the ATP Content Assay Kit (AKOP004M, Boxbio). Assays were performed according to the manufacturer’s instructions.

### O-dianisidine staining

*trim25*^+/+^ and *trim25*^−/−^ zebrafish larvae (38 h post-fertilization; n = 8) were placed in 60-mm cell culture dishes filled with 5 ml of egg water. They were incubated under normoxia (20% O_2_) or hypoxia (10% O_2_) for 10 h. The larvae were stained with O-dianisidine staining solution (Sigma-Aldrich O-dianisidine dissolved in 100% ethanol, 0.1 M sodium acetate, and 30% H_2_O_2_) for 15 min under light protection and washed with PBS. Finally, the larvae were placed on a layer of 3% methylcellulose M450 solution in a 60-mm cell culture dish and imaged using a Nikon TE2000-U microscope.

### ELISA

8-week-old mice were exposed to normoxia (21% O_2_) or hypoxia (10% O_2_) for 4 h. Mouse serum was collected and used to measure Epo concentration according to the manufacturer’s instructions for the ELISA kit (MEP00B, R&D Systems).

### Hypoxia treatment

The Ruskinn INVIVO2 I-400 workstation was used for hypoxia treatment of cells and animals (zebrafish and mice). Oxygen concentration and temperature were adjusted to specified values prior to hypoxia treatment. For hypoxia treatment of cells, cells were incubated in the hypoxia workstation (1% O_2_, 5% CO_2_, 37 °C) for the indicated time. For hypoxia treatment of adult zebrafish (3 mpf), *trim25*^+/+^ or *trim25*^−/−^ zebrafish with similar weight were placed in a conical flask containing 250 ml of water, adult zebrafish were then treated under hypoxic conditions (5% O_2_, 5% CO_2_, 28 °C). For hypoxia treatment of zebrafish larvae (3 dpf), zebrafish larvae were placed in 60-mm disposable cell culture dishes filled with 5 ml egg water were placed in the hypoxia workstation (2% O_2_, 5% CO_2_, 28 °C). To obtain the survival curve of adult zebrafish and zebrafish larvae under hypoxia, the number of dead zebrafish was counted, and the behavior of the zebrafish was closely observed, recorded, and photographed. For hypoxia treatment of adult mice (8 weeks old), each mouse was placed in a flask sealed with gauze and then placed in a hypoxia workstation (10% O_2_, 5% CO_2_, 25 °C). The behavior of the mice in the flasks was closely observed, recorded, photographed, and videotaped.

### Statistical analysis

For qRT-PCR comparing the differences between control and treated groups, an unpaired two-tailed Student’s *t* test was used. For the zebrafish survival curve analysis, the Kaplan-Meier method and log-rank test were used. All statistical analyses were performed with GraphPad Prism 8.0 software. Data with error bars represent the mean ± SD. A *p*-value <0.05 was considered significant. Data were derived from three independent reproducible experiments.

## Data availability

Any additional information in this paper is available from the corresponding author upon request.

## Supporting information

This article contains supporting information.

## Conflict of interest

The authors declare that they have no conflicts of interest with the contents of this article.
