# Supplementary material for: TRIM25 enhances hypoxia signaling by catalyzing K11-linked polyubiquitination and stabilization of HIF-α
Source: J Biol Chem. 2026 May 6;302(6):113125. doi: 10.1016/j.jbc.2026.113125 (PMC13241728; doi:10.1016/j.jbc.2026.113125)
Supplement: Supporting information [file mmc3.docx]

# Supporting information

**TRIM25 enhances hypoxia signaling by catalyzing K11-linked polyubiquitination and stabilization of HIF-α**

Ziyi Li ^1, 2, 3, 4^, Jun Li ^1, 2, 3, 4^, Zhi Li ^1, 2, 3, 4^, Rui Wang ^1, 2, 4^, Le Yuan ^1, 2, 3, 4^, Yanan Song ^1, 2, 3, 4^, Yanyi Wang ^1, 2, 3, 4^, Runkun Yan ^1, 2, 3, 4^, Fuxiang Lai ^1, 2, 3, 4^, Jing Wang ^1, 2, 3, 4^ and Wuhan Xiao ^1, 2, 3, 4, *^

Email: w-xiao@ihb.ac.cn

**This PDF file includes:**

Figure S1 to S6

Figure legends for figures S1 to S6

Video S1 and S2

Tables S1 and S2

## Figure S1


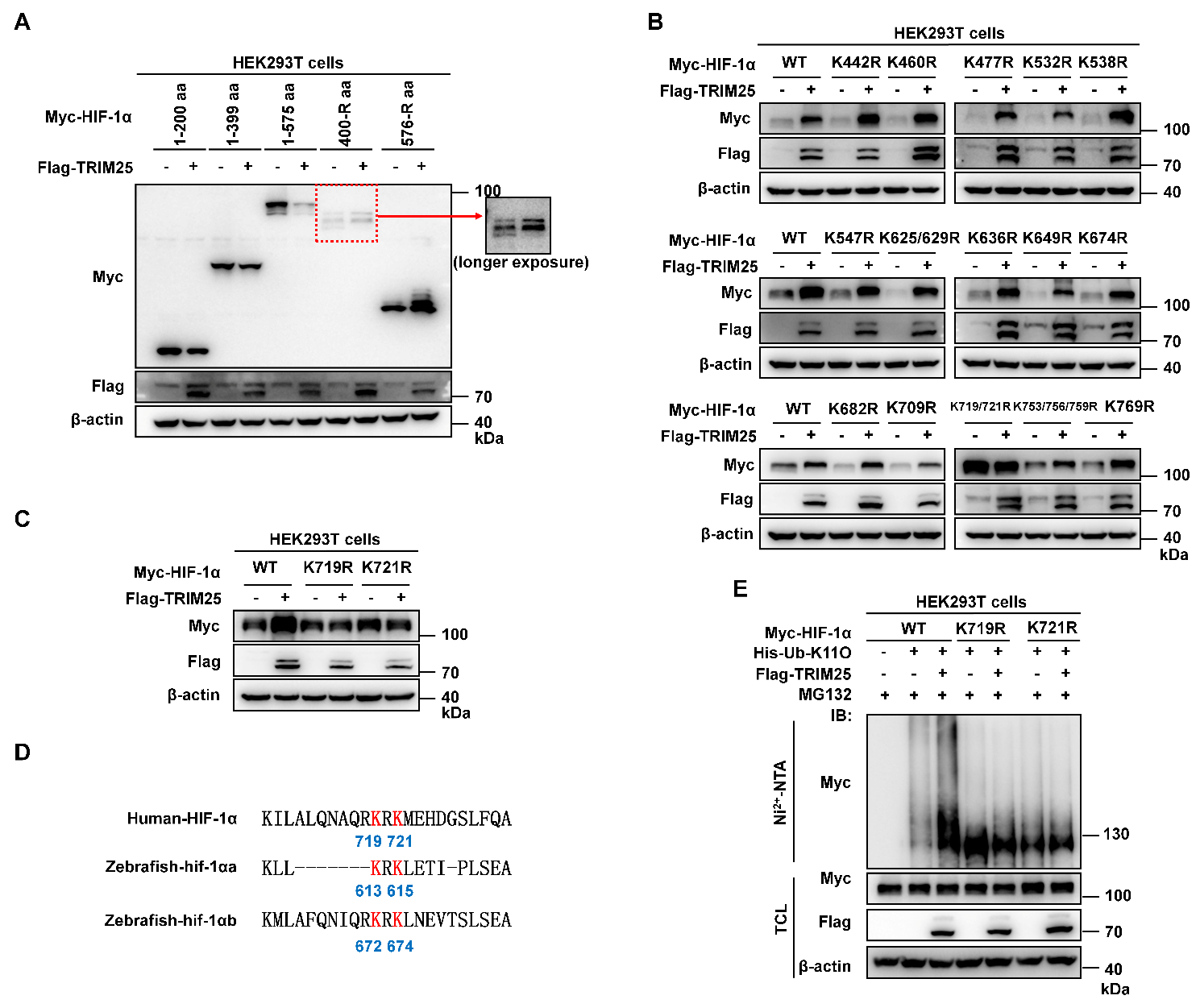


**Figure S1. TRIM25 targets HIF-1α at Lys719 and Lys721 for stabilization.**

**A** IB of the indicated proteins in HEK293T cells transfected with Flag-empty or Flag-TRIM25 together with the truncated Myc-HIF-1α constructs, 1-200 aa, 1-399 aa, 1-575 aa, 400-826 aa (400-R), and 576-826 aa (576-R) for 24 h. **B** IB of the indicated proteins in HEK293T cells transfected with Flag-empty or Flag-TRIM25 together with wild-type HIF-1α (WT), or its mutants with the indicated lysine mutated to arginine, including K442R, K460R, K477R, K532R, K538R, K547R, K625/629R, K636R, K649R, K674R, K682R, K709R, K719/721R, K753/756/759R, and K769R for 24 h. **C** IB of the indicated proteins in HEK293T cells transfected with Flag-empty or Flag-TRIM25 together with Myc-HIF-1α-WT, Myc-HIF-1α-K719R or Myc-HIF-1α-K721R for 24 h. **D** Alignment of the partial amino acid sequences of human HIF-1α, zebrafish hif-1αa and zebrafish hif-1αb. **E** IB for K11-linked ubiquitination of HIF-1α, and its mutants HIF-1α-K719R and HIF-1α-K721R in HEK293T cells.

## Figure S2


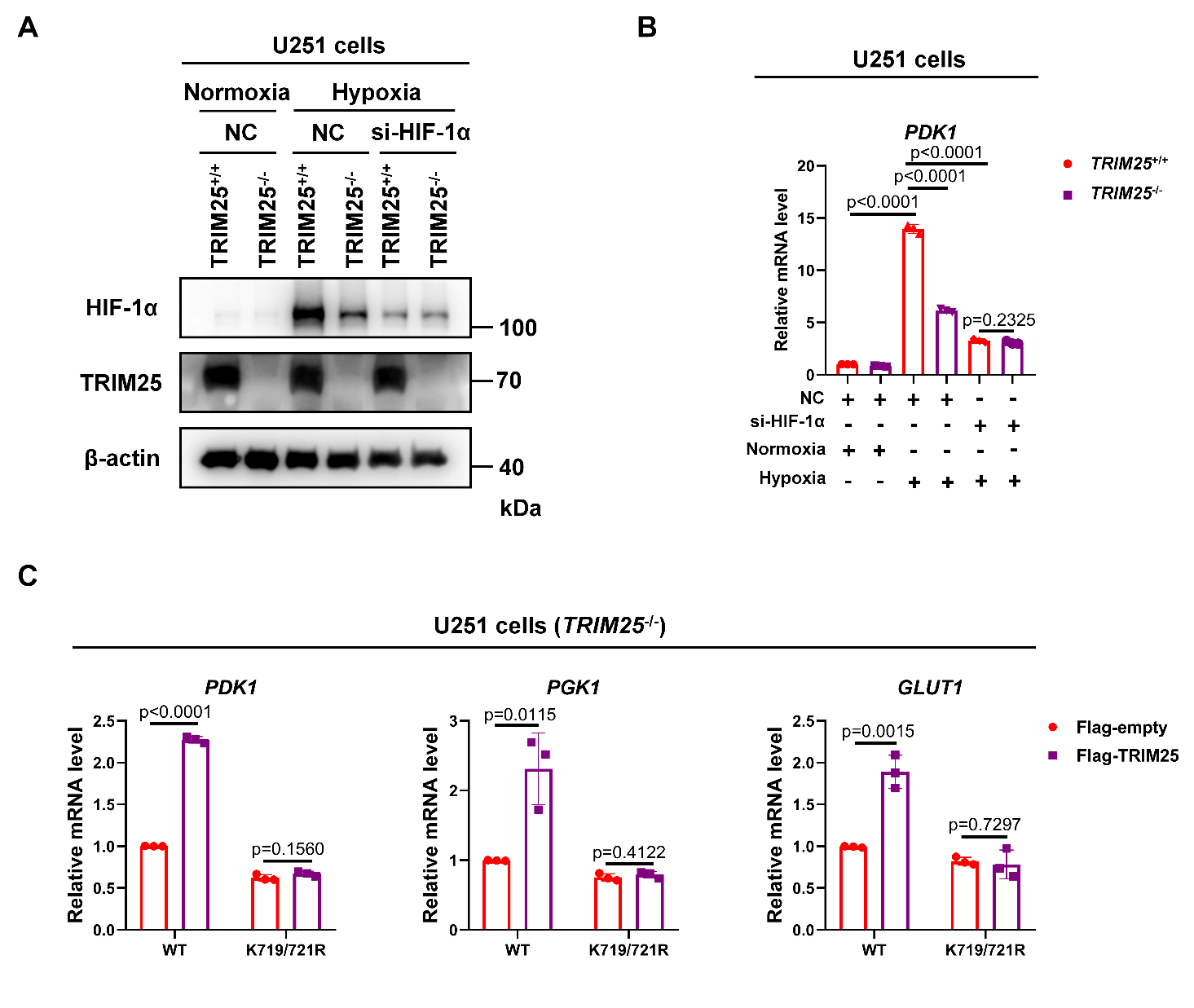


**Figure S2. TRIM25 targets HIF-1α at Lys719 and Lys721 to promote hypoxia signaling.**

**A** IB of HIF-1α in *TRIM25*^+/+^ and *TRIM25*^-/-^ U251 cells after HIF-1α knockdown by siRNA (or NC control) under normoxia and hypoxia. NC, negative control. **B** qPCR of *PDK1* mRNA in *TRIM25*^+/+^ and *TRIM25*^-/-^ U251 cells with and without HIF-1α knockdown by siRNA. **C** qPCR of *PDK1*, *PGK1* and *GLUT1* mRNA in *TRIM25*^-/-^ U251 cells transfected with Myc-HIF-1α-WT or Myc-HIF-1α-K719/721R under normoxia. Data in (**B**) and (**C**) are shown as mean ± SD; statistical significance was determined by unpaired two-tailed Student’s t-test; each point represents a technical replicate of a representative experiment from three independent experiments.

## Figure S3


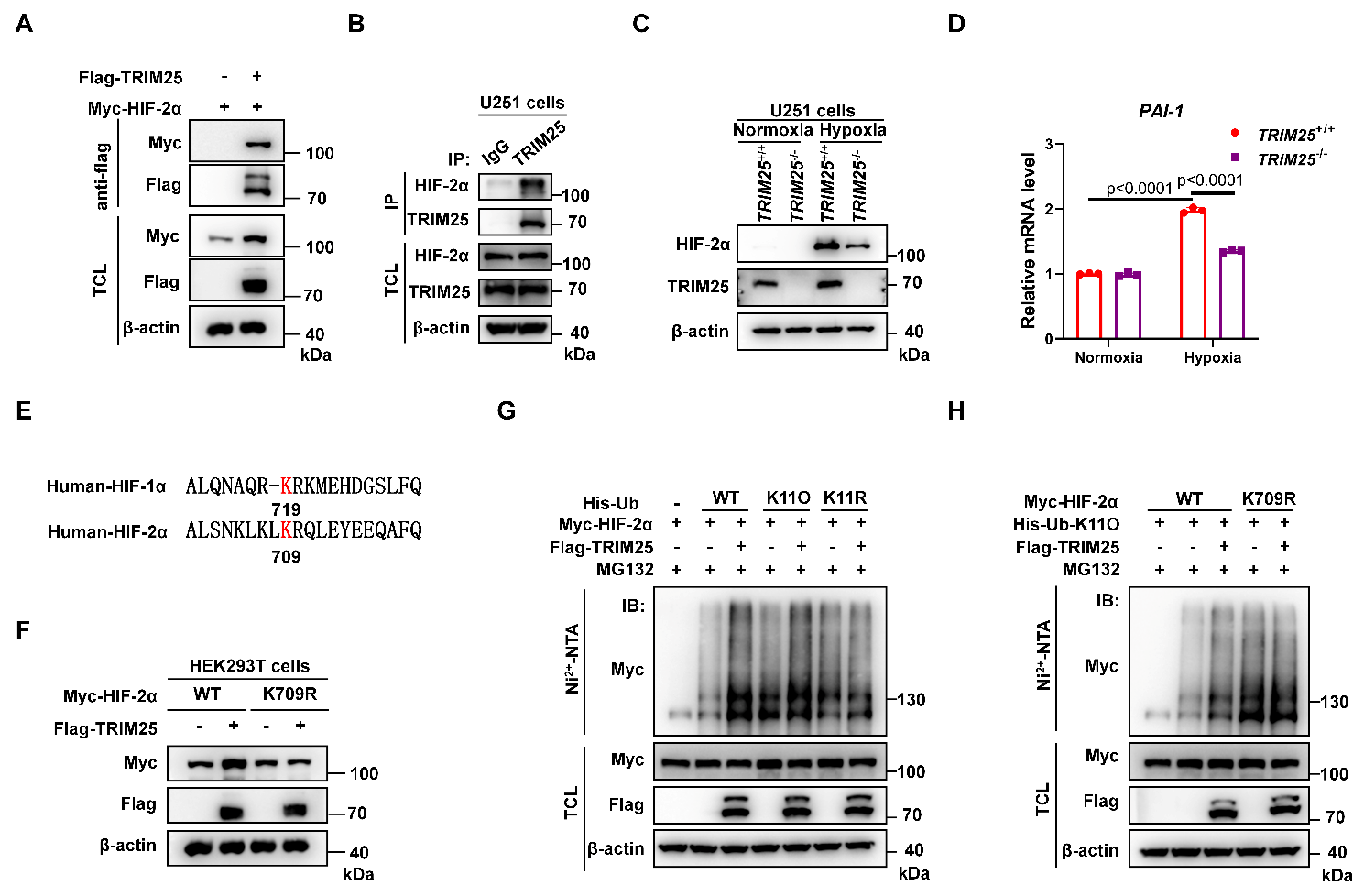


**Figure S3. TRIM25 targets HIF-2α at Lys709 to catalyze K11-linked polyubiquitination, resulting in stabilization.**

**A** Co-IP of ectopically expressed TRIM25 interacting with ectopically expressed HIF-2α in HEK293T cells. **B** Co-IP of endogenous TRIM25 interacting with endogenous HIF-2α in U251 cells cultured under hypoxia (1% O_2_) for 4 h. **C** IB of endogenous HIF-2α in wild-type (*TRIM25*^+/+^) and *TRIM25*-deficient (*TRIM25*^-/-^) U251 cells cultured under normoxia (21% O_2_) or hypoxia (1% O_2_) for 4 h. **D** qPCR analysis of *PAI-1* mRNA in wild-type (*TRIM25*^+/+^) and *TRIM25*-deficient (*TRIM25*^-/-^) U251 cells under normoxia (21% O_2_) or hypoxia (1% O_2_) for 16 h. **E** Alignment of the partial amino acid sequences of human HIF-1α and HIF-2α. **F** IB of the indicated proteins in HEK293T cells transfected with Myc-*HIF-2α*-WT or its mutant Myc-*HIF*-2α*-*K709R together with Flag-empty vector or Flag-*TRIM25* for 24 h. **G** IB of HIF-2α ubiquitination in HEK293T cells transfected with Myc-*HIF-2α*, Flag-empty vector or Flag-*TRIM25* together with His-Ub-WT, His-Ub-K11O or His-Ub-K11R for 20 h, then treated with MG132 (20 μM) for 4 h. **H** IB of HIF-2α and its mutant HIF-2α*-*K709R ubiquitination in HEK293T cells transfected with His-Ub-K11O, Myc-*HIF*-2α*-*WT or Myc-*HIF*-2α*-*K709R together with Flag-empty vector or Flag-*TRIM25* for 20 h, then treated with MG132 (20 μM) for 4 h. Data in (***D***) are shown as mean ± SD; statistical significance was determined by unpaired two-tailed Student’s t-test; each point represents a technical replicate of a representative experiment from three independent experiments.

## Figure S4


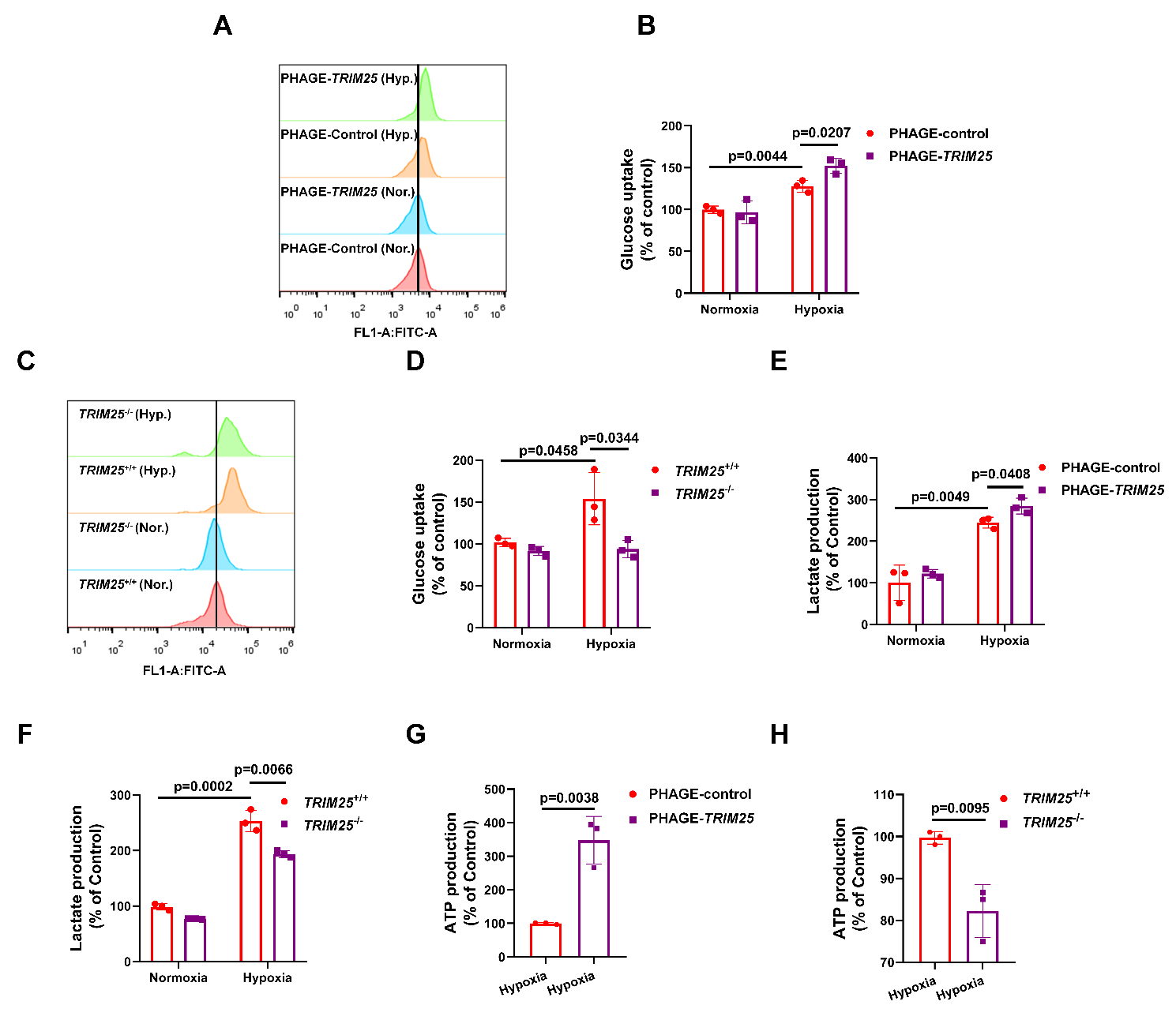


**Figure S4.** **TRIM25 facilitates hypoxia adaptation.**

**A, B** Flow cytometric analysis of glucose uptake in *TRIM25*^-/-^ U251 cells reconstituted with PHAGE-control or PHAGE-*TRIM25* under normoxia (21% O_2_) or hypoxia (1% O_2_). **C, D** Flow cytometric analysis of glucose uptake in wild-type (*TRIM25*^+/+^) and *TRIM25*-deficient (*TRIM25*^-/-^) U251 cells under normoxia (21% O_2_) or hypoxia (1% O_2_). **E** Lactate production in *TRIM25*^-/-^ U251 cells reconstituted with PHAGE-control or PHAGE-*TRIM25* under normoxia (21% O_2_) or hypoxia (1% O_2_) for 24 h. **F** Lactate production in wild-type (*TRIM25*^+/+^) and *TRIM25*-deficient (*TRIM25*^-/-^) U251 cells under normoxia (21% O_2_) or hypoxia (1% O_2_) for 24 h. **G** ATP production in *TRIM25*^-/-^ U251 cells reconstituted with PHAGE-control or PHAGE-*TRIM25* under hypoxia (1% O_2_) for 24 h. **H** ATP production in wild-type (*TRIM25*^+/+^) and *TRIM25*-deficient (*TRIM25*^-/-^) U251 cells under hypoxia (1% O_2_) for 24 h. Data in [(***B***), and (***D***) to (***H***)] are shown as mean ± SD; statistical significance was determined by unpaired two-tailed Student’s t-test; each point represents a biological replicate of a representative experiment from three independent experiments.

## Figure S5


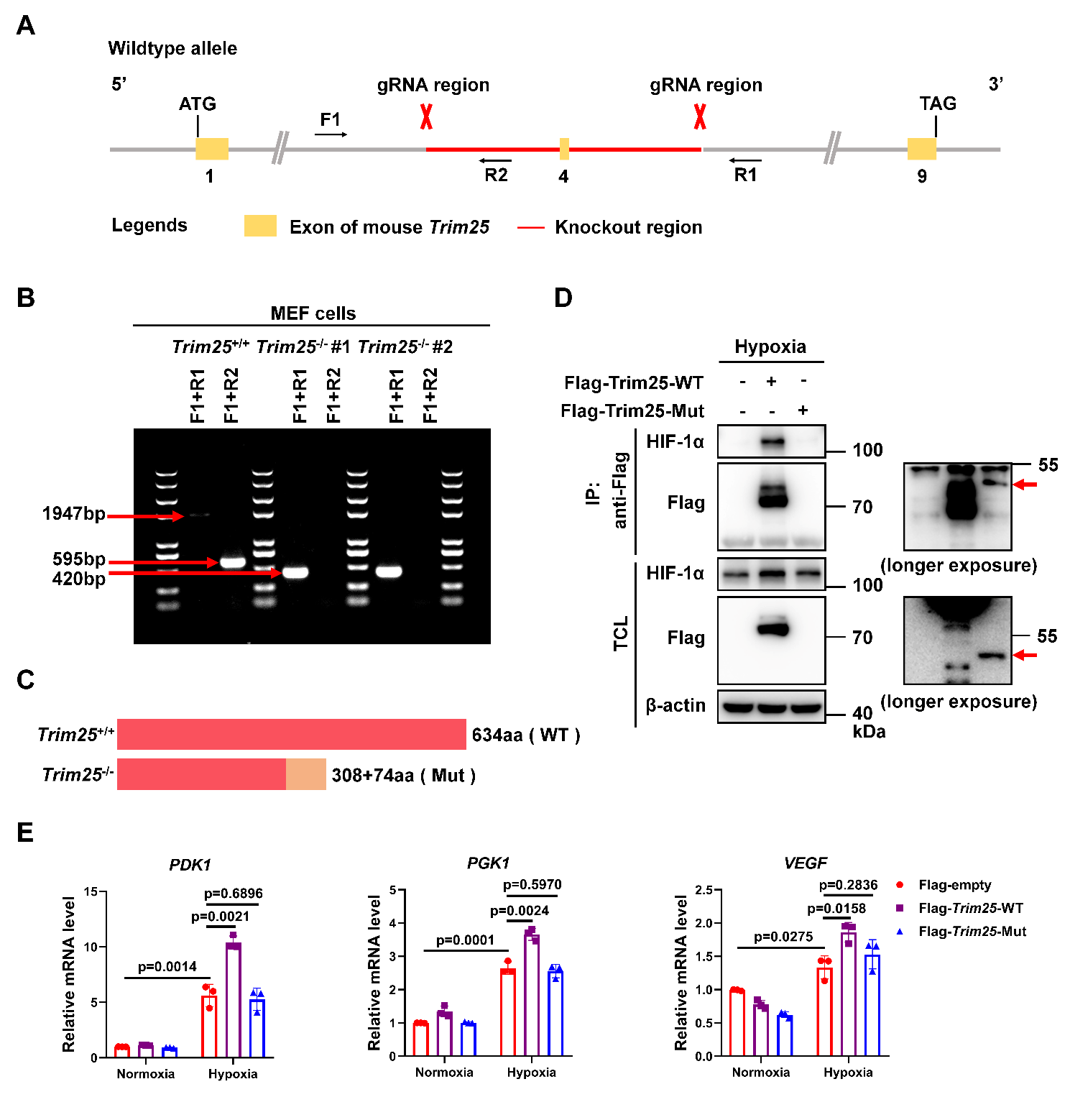


**Figure S5. CRISPR/Cas9-mediated generation of *Trim25*^-/-^ mouse.**

**A** Diagram of the target site and deletion in the mouse *Trim25*. **B** Identification of MEF cell genotypes by PCR. **C** The predicted protein product of *Trim25* in the mutant (Mut) and wild-type siblings (WT). **D** Co-IP of endogenous HIF-1α interacting with Flag-Trim25-WT or Flag-Trim25-Mut in HEK293T cells cultured under hypoxia (1% O_2_) for 4 h. **E** qPCR analysis of *PDK1*, *PGK1* and *VEGF* mRNA in *TRIM25*-deficient U251 cells (*TRIM25*^-/-^) transfected with Flag-empty vector, Flag-*Trim25*-WT or Flag-*Trim25*-Mut and cultured under normoxia (21% O_2_) or hypoxia (1% O_2_) for 16 h. Data in (***E***) are shown as mean ± SD; statistical significance was determined by unpaired two-tailed Student’s t-test; each point represents a technical replicate of a representative experiment from three independent experiments.

## Figure S6


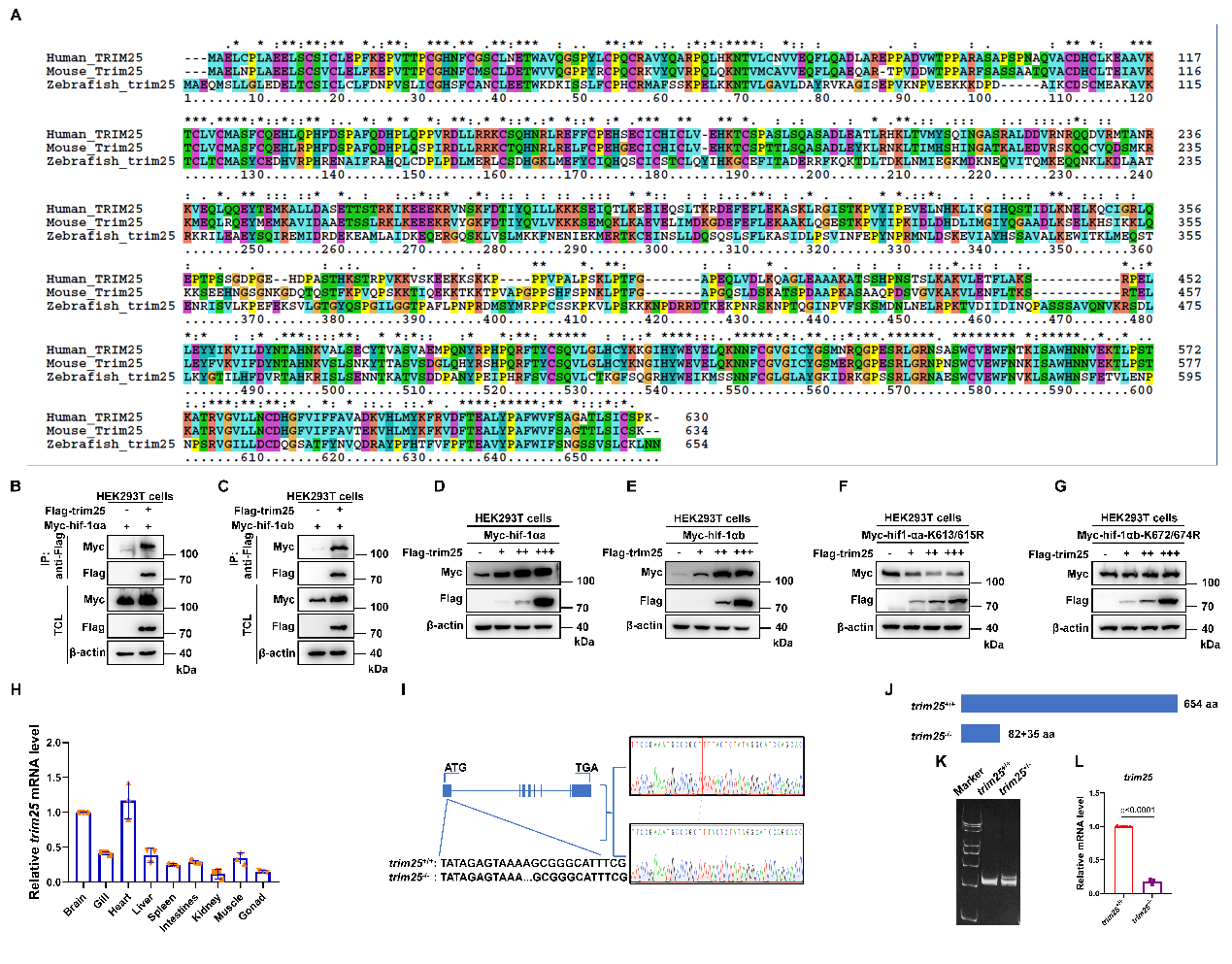


**Figure S6. Zebrafish trim25 binds to hif-1α and stabilizes hif-1α protein level.**

**A** Alignment of TRIM25 amino acid sequences from human, mouse and zebrafish. **B** Co-IP of Flag-trim25 interacting with Myc-hif-1αa in HEK293T cells. **C** Co-IP of Flag-trim25 interacting with Myc-hif-1αb in HEK293T cells. **D** IB of exogenous Myc-hif-1αa expression in HEK293T cells transfected with Myc-*hif-1αa* together with Flag-empty or increasing amounts of Flag-*trim25* expression plasmids. **E** IB of exogenous Myc-hif-1αb expression in HEK293T cells transfected with Myc-*hif-1αb* together with Flag-empty or increasing amounts of Flag-*trim25* expression plasmids. **F** IB of exogenous Myc-hif-1αa-K613/615R expression in HEK293T cells transfected with Myc-*hif-1αa*-K613/615R together with Flag-empty or increasing amounts of Flag-*trim25* expression plasmids. **G** IB of exogenous Myc-hif-1αb-K672/674R expression in HEK293T cells transfected with Myc-*hif-1αb*-K672/674R together with Flag-empty or increasing amounts of Flag-*trim25* expression plasmids. **H** qPCR analysis of *trim25* mRNA in brain, gill, heart, liver, spleen, intestines, kidney, muscle and gonad of adult wild-type zebrafish (3 mpf). **I** Diagram of the sequence information in *trim25*^+/+^ and *trim25*^-/-^ zebrafish. One base pair was deleted in exon 1 of *trim25* in the mutant, resulting in a reading frame shift. **J** The predicted protein product of *trim25* in the mutant and wild-type siblings. **K** Verification of the efficiency of CRISPR/Cas9-mediated *trim25* disruption by heteroduplex mobility assay (HMA). **L** qPCR analysis of *trim25* mRNA in the wild-type or *trim25*-deficient zebrafish larvae (3 dpf). Data in [(***H***), and (***L***)] are shown as mean ± SD; statistical significance was determined by unpaired two-tailed Student’s t-test; each point represents a technical replicate of a representative experiment from three independent experiments.

**Video S1** *Trim25*^-/-^ mouse (right) and their wild-type siblings (*Trim25*^+/+^) (left) set in the hypoxia workstation (10% O_2_) for 2 h.

**Video S2** *trim25* ^-/-^ zebrafish (right) and their wild-type siblings (*trim25*^+/+^) (left) set in the hypoxia workstation (5% O_2_) for 4 h.

## Table S1. Reagents and resource

| **REAGENT or RESOURCE** | **Source** | **Identifier** |
| --- | --- | --- |
| **Antibodies** | | |
| Mouse monoclonal anti-Myc | Santa Cruz | Cat#sc-40 |
| Mouse monoclonal anti-Flag | Sigma-Aldrich | Cat#F1804 |
| Rabbit monoclonal anti-β-actin | ABclonal | Cat#AC026 |
| Rabbit monoclonal anti-HIF-1α | Cell Signaling Technology | Cat#36169 |
| Rabbit monoclonal anti-HIF-2α | Cell Signaling Technology | Cat#7096 |
| Rabbit monoclonal anti-Histone H3 | Cell Signaling Technology | Cat#4499 |
| Mouse monoclonal anti-α-tubulin | Thermo Fisher | Cat#62204 |
| normal rabbit IgG | Cell Signaling Technology | Cat#2729 |
| Rabbit monoclonal anti-Ubiquitin | Cell Signaling Technology | Cat#3936 |
| Rabbit polyclonal anti-TRIM25 | abclone | Cat# A12938 |
| **Chemicals, peptides, and recombinant proteins** | | |
| DMSO | Sigma-Aldrich | Cat#D2650 |
| MG-132 | Sigma-Aldrich | Cat#474790 |
| FG4592 | Selleck | Cat#S1007 |
| [Cycloheximide](https://www.sciencedirect.com/topics/biochemistry-genetics-and-molecular-biology/cycloheximide) (CHX) | MedChemExpress | Cat#HY-12320 |
| CoCl_2_ | Sigma-Aldrich | Cat#C8661 |
| DAPI | Thermo Fisher | Cat#62248 |
| TransZol Up | TransGen Biotech | Cat# ET111-01-V2 |
| Transfection Reagent VigoFect | Vigorous Biotech | Cat#T001 |
| Protease Inhibitor Cocktail | Bimake | Cat#B14001 |
| 2-NBDG | MedChemExpress | Cat#HY-116215 |
| β-Mercaptoethanol | GEN-VIEW | Cat#GM195 |
| **Critical commercial assays** | | |
| One-Step gDNA Removal and cDNA Synthesis SuperMix | TransGen Biotech | Cat# AT311-02 |
| SYBR Green qRCR Mix (High ROX) | Monad Biotech | Cat# MQ10301S |
| Anti-Flag Affinity Gel | Yeasen | Cat# 20585ES08 |
| Protein G Sepharose 4 Fast Flow | Cytiva | Cat#17061801 |
| Mouse Epo ELISA kit | Promoter | Cat#MEP00B |
| L-Lactic Acid (L-LA) Content Assay Kit | Boxbio | Cat#AKAC001C |
| ATP Content Assay Kit | Boxbio | Cat#AKOP004M |
| **Experimental models: Cell lines** | | |
| HEK293T | ATCC | Cat #CRL-11268 |
| U251 | Chinese Academy of Sciences (Shanghai, China) | Cat #SCSP-559 |
| H1299 | ATCC | Cat #CRL-5803 |
| *Trim25^-/-^* MEFs | This paper | N/A |
| MEFs | This paper | N/A |
| *TRIM25*-knockout U251 | This paper | N/A |
| **Experimental models: Organisms/strains** | | |
| *C57BL/6J-Trim25^em1C^/Cya* | Cyagen Biosciences | KOCMP-217069-Trim25-B6J-VB |
| Zebrafish/Strain AB | Chinese Zebrafish Resource Center | N/A |
| Zebrafish *trim25* ^ihblzy01^ | This paper | [https://zfin.org/ZDB-ALT-240708-7](https://zfin.org/ZDB-ALT-230809-2%20) |
| **Oligonucleotides** | | |
| The zebrafish-*trim25*-gRNA targeting sequence:  5’-GGATGCCTATAGAGTAAAAG-3’ | This paper | N/A |
| The Human-*TRIM25*-gRNA targeting sequence:  5’-GCACGACAGCTCCTCGGCCA-3' | This paper | N/A |
| Primers for RT-PCR, see Supplementary Table 2 | This paper | N/A |
| **Recombinant DNA** | | |
| pCMV-Myc | Clontech | Cat #K6003-1 |
| pCMV-Flag | This study | N/A |
| pCMV-Flag-TRIM25 | This study | N/A |
| pCMV-Flag-TRIM25-ΔRING | This study | N/A |
| pCMV-Flag-TRIM25-C/S | This study | N/A |
| pCMV-Myc-HIF-1α | This study | N/A |
| pCMV-HA-HIF-1α-DM | This study | N/A |
| pCMV-Myc-HIF-1α-1-200 | This study | N/A |
| pCMV-Myc-HIF-1α-1-399 | This study | N/A |
| pCMV-Myc-HIF-1α-1-575 | This study | N/A |
| pCMV-Myc-HIF-1α-400-R | This study | N/A |
| pCMV-Myc-HIF-1α-576-R | This study | N/A |
| His-ubiquitin | This study | N/A |
| His-ubiquitin-K6R | This study | N/A |
| His-ubiquitin-K11R | This study | N/A |
| His-ubiquitin-K27R | This study | N/A |
| His-ubiquitin-K29R | This study | N/A |
| His-ubiquitin-K33R | This study | N/A |
| His-ubiquitin-K48R | This study | N/A |
| His-ubiquitin-K63R | This study | N/A |
| His-ubiquitin-K11O | This study | N/A |
| pCMV-Myc-HIF-1α-K442R | This study | N/A |
| pCMV-Myc-HIF-1α-K460R | This study | N/A |
| pCMV-Myc-HIF-1α-K477R | This study | N/A |
| pCMV-Myc-HIF-1α-K532R | This study | N/A |
| pCMV-Myc-HIF-1α-K538R | This study | N/A |
| pCMV-Myc-HIF-1α-K547R | This study | N/A |
| pCMV-Myc-HIF-1α-K625/629R | This study | N/A |
| pCMV-Myc-HIF-1α-K636R | This study | N/A |
| pCMV-Myc-HIF-1α-K649R | This study | N/A |
| pCMV-Myc-HIF-1α-K674R | This study | N/A |
| pCMV-Myc-HIF-1α-K682R | This study | N/A |
| pCMV-Myc-HIF-1α-K709R | This study | N/A |
| pCMV-Myc-HIF-1α-K719/721R | This study | N/A |
| pCMV-Myc-HIF-1α-K753/756/759R | This study | N/A |
| pCMV-Myc-HIF-1α-K769R | This study | N/A |
| pCMV-Myc-HIF-1α-K719R | This study | N/A |
| pCMV-Myc-HIF-1α-K721R | This study | N/A |
| pCMV-Myc-HIF-2α | This study | N/A |
| pCMV-Myc-HIF-2α-K709R | This study | N/A |
| pCMV-Flag-trim25 | This study | N/A |
| pCMV-Myc-hif1αa | This study | N/A |
| pCMV-Myc-hif1αb | This study | N/A |
| pCMV-Myc-hif1αa-K613/615R | This study | N/A |
| pCMV-Myc-hif-1αb-K672/674R | This study | N/A |
| pCMV-Flag-Trim25 | This study | N/A |
| pCMV-Flag-Trim25-KO | This study | N/A |
| pGEX-4T-1 | This study | N/A |
| pGEX-4T-1-HIF-1α | This study | N/A |
| pet-32a-TRIM25 | This study | N/A |
| **Software and algorithms** | | |
| ImageJ | Software | <https://imagej.nih.gov/ij/> |
| GraphPad Prism | Software | https://www.graphpad.com /scientific-software/prism/ |
| FlowJo | Software | <https://www.flowjo.com/> |

## Table S2. The primers for qRT-PCR

| **Primers** | **Sequence (5’ to 3’)** |
| --- | --- |
| Zebrafish-*β-actin*-RT-F | TACAATGAGCTCCGTGTTGC |
| Zebrafish-*β-actin*-RT-R | ACATACAATGGCAGGGGTGTT |
| Zebrafish-*trim25*-RT-F | GCGTCTCTGTTCCGATCATG |
| Zebrafish-*trim25*-RT-R | GGGTGGCAGCTAGATCCTTT |
| Zebrafish-*phd3*-RT-F | CCTGGAAATGGAGCTGGATA |
| Zebrafish-*phd3*-RT-R | CCGGTCAAATAAAGGCTCAA |
| Zebrafish-*epo*-RT-F | GCATCAGACAAGTGCTGCG |
| Zebrafish-*epo*-RT-R | AGACAGGTGCATTGGCGAG |
| Zebrafish-*pdk1*-RT-F | TGAACCAGCACACTCTTCTG |
| Zebrafish-*pdk1*-RT-R | AGCATCTTTTACCACATCCG |
| Zebrafish-*vegf*-RT-F | GCATCAGACAAGTGCTGCG |
| Zebrafish-*vegf*-RT-R | AGACAGGTGCATTGGCGAG |
| Zebrafish-*glut1*-RT-F | GTGATTGGGTCCTTGCAGTT |
| Zebrafish-*glut1*-RT-R | CTGAGAAGGAGCCGAGAATG |
| Mouse-*β-actin*-RT-F | CCTGAGCGCAAGTACTCTGTGT |
| Mouse-*β-actin*-RT-R | GCTGATCCACATCTGCTGGAA |
| Mouse-*Glut1*-RT-F | ATCCCAGCAGCAAGAAGGTGA |
| Mouse-*Glut1*-RT-R | TGGTGGATGGGATGGGCTCTCC |
| Mouse-*Vegf*-RT-F | ATGCCAAGTGGTCCCAGGCTGC |
| Mouse-*Vegf*-RT-R | ATCGGACGGCAGTAGCTTCGC |
| Mouse-*Pgk1*-RT-F | ATTCTGCTTGGACAATGGAGC |
| Mouse-*Pgk1*-RT-R | AGGCATGGGAACACCATCA |
| Mouse-*Epo*-RT-F | AATGGAGGTGGAAGAACAGG |
| Mouse-*Epo*-RT-R | ACCCGAAGCAGTGAAGTGA |
| Human-*18S rRNA*-RT-F | CGGCGACGACCCATTCGAAC |
| Human-*18S rRNA*-RT-R | GAATCGAACCCTGATTCCCCGTC |
| Human-*GLUT1*-RT-F | CGGGCCAAGAGTGTGCTAAA |
| Human-*GLUT1*-RT-R | TGACGATACCGGAGCCAATG |
| Human-*PDK1*-RT-F | ACCAGGACAGCCAATACAAG |
| Human-*PDK1*-RT-R | CCTCGGTCACTCATCTTCAC |
| Human-*PGK1*-RT-F | TGGCTTCTGGCATACCTGCT |
| Human-*PGK1*-RT-R | GCTGCTTTCAGGACCACAGCT |
| Human-*VEGF*-RT-F | CTTGCCTTGCTGCTCTAC |
| Human-*VEGF*-RT-R | TGGCTTGAAGATGTACTCG |
| Human-*EPO*-RT-F | ATCACGACGGGCTGTGCTGAACAC |
| Human-*EPO*-RT-R | GGGAGATGGCTTCCTTCTGGGCTC3 |
| Human-*PAI-1*-RT-F | AAAGGCAACATGACCAGGCT |
| Human-*PAI-1*-RT-R | GGGAGAACTTGGGCAGAACC |
